# Influence of gradient Ni incorporation on the structural, surface, and electrochemical properties of LiCoO_2_ thin-film cathodes

**DOI:** 10.1039/d6ra06673a

**Published:** 2026-10-22

**Authors:** Z. Benzarti, C. Vicente, J. D. Castro, S. Cruz, S. Carvalho

**Affiliations:** a CEMMPRE, ARISE, Mechanical Engineering Department, University of Coimbra 3030-788 Coimbra Portugal zohra.benzarti@dem.uc.pt; b Laboratory of Multifunctional Materials and Applications (LaMMA), Faculty of Sciences of Sfax, University of Sfax BP 1171 Sfax 3000 Tunisia; c Environmental Engineering Department, University of Coimbra 3030-790 Coimbra Portugal; d IPN—LED & MAT—Instituto Pedro Nunes, Laboratory of Tests, Wear and Materials Rua Pedro Nunes 3030-199 Coimbra Portugal

## Abstract

Layered LiCoO_2_ thin-film (LCO) cathodes are attractive for lithium-ion microbatteries, but their use is constrained by structural instability, modest rate capability, and progressive capacity loss. In this work, we show that spatially graded Ni incorporation, achieved *via* controlled RF/DC co-sputtering, provides a promising strategy to tune structural and electrochemical limitations in layered LiNi_*x*_Co_1−*x*_O_2_ (LNCO) thin film cathodes. Three architectures were developed: LCO, uniformly Ni-incorporated LNCO-0.25A, and gradient Ni-incorporated LNCO-G, the latter obtained by progressively increasing the Ni target current from 0.20 to 0.40 A during deposition, producing a depth-dependent Ni distribution. XRD and Raman analyses show that Ni incorporation is accompanied by sharper reflections and reduced Raman peak broadening, consistent with improved local structural order. Cross-sectional HAADF-STEM and STEM–EDS mapping confirm a dense microstructure and a continuous Ni gradient increasing progressively from the substrate interface toward the coating surface. Depth-resolved XPS shows that, although all films develop surface carbonate species upon air exposure, LNCO-G exhibits a reduced hydroxyl/carbonate contribution compared with LNCO-0.25A, which is consistent with a less hydrated near-surface chemistry. Electrochemically, LNCO-G delivers higher current densities (≈3–5× increase) and more defined high-voltage redox features than the uniformly incorporated counterpart. In a preliminary 10-cycle diagnostic test under an aggressive 1.0–4.9 V window, LNCO-G achieves an initial discharge capacity of ∼100–120 mAh g^−1^ at 0.5C and retains ∼55% of its capacity. This short, aggressive protocol was intentionally chosen as a materials-level screening test to amplify differences in polarization and early degradation among the three architectures. Post-cycling EIS shows a 59% reduction in charge-transfer resistance (67.7 Ω *vs.* 164.3 Ω) and a lower Warburg coefficient (21.3 *vs.* 44.1 Ω s^−1/2^) relative to LNCO-0.25A. These results demonstrate that compositional Ni grading is associated with a measurable performance advantage over the uniform incorporation strategy studied here, although the comparison is not composition-matched and therefore reflects combined effects of Ni content, distribution, and secondary-phase fraction. These findings identify spatial Ni grading as a promising route for tuning coupled structural, interfacial, and kinetic properties in sputtered LiCoO_2_-based thin-film cathodes.

## Abstract 2

Layered LiCoO_2_ thin-film (LCO) cathodes are attractive for lithium-ion microbatteries, but their use is constrained by structural instability, modest rate capability, and progressive capacity loss.

## Graphical abstract

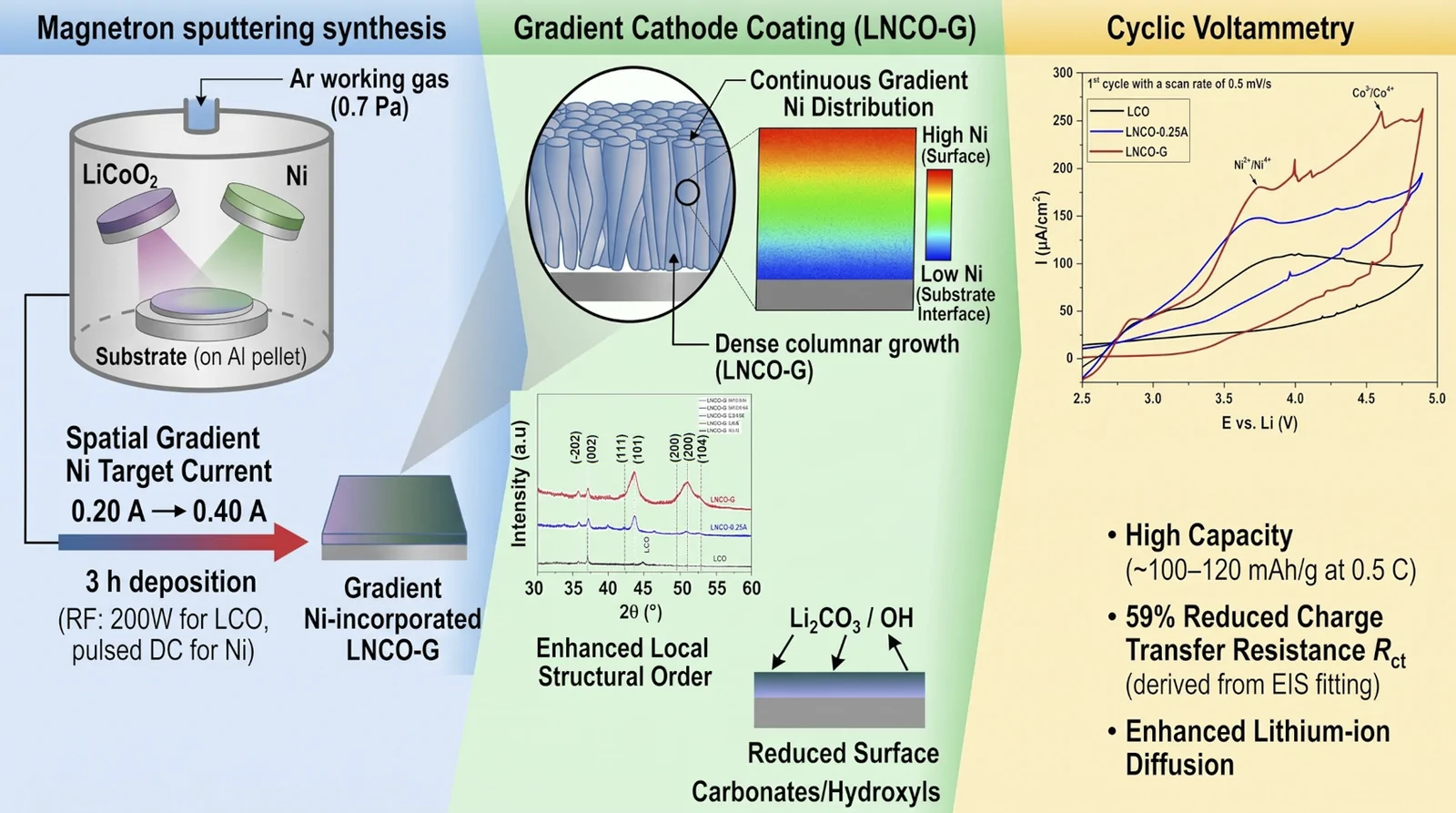

## 1. Introduction

Lithium-ion batteries (LIBs) have become the dominant energy-storage technology for portable electronics, electric vehicles, and renewable energy storage systems, owing to their high energy density, long cycle life, and favorable safety profile.^1–3^ However, despite continuous progress in electrode design, the energy density of LIBs remains fundamentally constrained by the performance of the cathode materials, which directly governs battery cost, thermal stability, and achievable operating voltage window.^1,2^ Consequently, the development of advanced cathode materials capable of sustaining higher voltages while maintaining structural integrity is a central challenge in next-generation battery research.

Among commercially deployed cathodes, lithium cobalt oxide (LiCoO_2_, LCO), continues to dominate portable device applications due to its high electronic conductivity, stable O_3_-type layered structure,^4,5^ and reliable cycling behavior. Nevertheless, LCO exhibits intrinsic limitations that restrict its practical capacity to approximately 140 mAh g^−1^. These limitations arise primarily from structural instability at voltages above 4.2 V,^6^ where excessive lithium extraction induces irreversible oxygen loss and phase transitions from the O_3_ phase to highly unstable H_1−3_ structures.^7,8^ Such transformations generate mechanical stress, particle cracking, and accelerated capacity fading, thereby limiting the applicability of LCO in high-energy-density systems.^9–11^ These voltage-dependent failure pathways are broadly observed across layered cathode systems, where structural breakdown and interfacial instability proceed concurrently during deep delithiation.^12^

To address these challenges, various material-modification strategies, including crystal facet engineering,^13^ interphase design,^14^ and transition metal substitution,^15^ have been developed to stabilize cathode structures and electrode–electrolyte interfaces.

In particular, the introduction of nickel has shown promise in mitigating high-voltage phase transitions, reducing interfacial resistance, and modifying cation distribution. In bulk powder systems, compositional gradient strategies typically utilize a Ni-rich core and a Ni-poor surface to reduce interfacial parasitic reactions, as demonstrated by Xu *et al.*^16^ At the atomic level, partial substitution of Co^3+^ by Ni^2+^, to form layered LiNi_*x*_Co_1−*x*_O_2_ (LNCO) solid solutions, may induce lattice expansion and influence cation ordering tendencies.^17^

In thin-film planar architectures, spatial Ni distribution affects physical and electrochemical properties differently from average compositional changes. Because Ni^2+^ and Co^3+^ differ in ionic radius, Ni incorporation into the layered oxide framework may generate local lattice distortion and composition-dependent structural environments. A depth-dependent Ni distribution could therefore produce a progressive variation in lattice dimensions and growth-induced stress through the coating thickness than uniform Ni incorporation, potentially limiting localized structural disorder. Compositional gradients have been reported to moderate mechanical and structural heterogeneity in layered oxide cathodes, although the corresponding strain distribution is not measured directly in the present study.^9,18,19^

Furthermore, top-surface Ni enrichment in thin-film configurations modifies interfacial reaction pathways by modifying the relative formation of oxide, hydroxide, and carbonate species. In particular, the development of an oxide-dominated Ni-containing surface environment could reduce the accumulation of hydroxyl-rich species compared with the uniformly Ni-incorporated coating. On this basis, the working hypothesis of this study is that gradient Ni incorporation in sputtered LiCoO_2_ films regulates both structural ordering and near-surface chemistry, thereby improving interfacial charge transfer and Li^+^ transport.

Beyond composition, crystallinity plays a decisive role in determining the electrochemical performance of LiCoO_2_-based cathodes, particularly in thin-film architectures, where well-ordered crystal structure facilitates Li^+^ diffusion and enhance transport kinetics. Therefore, achieving high crystallinity is essential for improving rate capability in miniaturized energy-storage devices.^20^ In this context, RF magnetron sputtering has emerged as a versatile technique for fabricating thin-film cathodes with controlled thickness, composition, and microstructure.^21^ Compared to conventional synthesis routes, sputtering enables precise compositional control and can produce dense films with relatively high crystallinity, making it well suited for microbatteries and integrated electronic applications.^22^ Importantly, sputtering also enables the fabrication of compositional graded thin films by dynamically varying deposition parameters during growth.^23^ Although gradient structures in powder systems can mitigate surface reactivity and mechanical degradation,^18,19^ applying compositional gradients to LiCoO_2_-based thin-film cathodes remains underexplored.

Despite these advantages, the application of compositional gradient strategies to LiCoO_2_-based thin-film cathodes remains relatively underexplored, particularly with respect to their influence on crystal structure, surface chemistry, and electrochemical reversibility.

In this work, we investigate the effect of nickel incorporation strategy on the structural, morphological, and electrochemical properties of RF-sputtered LiCoO_2_-based thin films. Three architectures were examined: LCO, uniformly Ni-incorporated LiCoO_2_ (LNCO-0.25A) prepared at a constant Ni target current of 0.25 A, and gradient Ni-incorporated LiCoO_2_ (LNCO-G) obtained by progressively increasing the Ni target current from 0.20 to 0.40 A during deposition. A combination of X-ray diffraction (XRD), Raman spectroscopy, scanning electron microscopy with energy-dispersive spectroscopy (SEM/EDS), X-ray photoelectron spectroscopy (XPS), cyclic voltammetry (CV), and galvanostatic charge–discharge (GCD) measurements is employed to elucidate the influence of Ni incorporation on structure and electrochemical behavior. By comparing uniform and gradient Ni incorporation under comparable thin-film conditions, this study aims to clarify how spatial compositional variations affect crystallinity, interfacial chemistry, and electrochemical response. The findings highlight the potential of compositional gradient engineering as a design strategy for improving the performance of LiCoO_2_-based thin-film cathodes.

## 2. Materials and methods

### 2.1. Sample deposition

LiCoO_2_-based thin films were deposited by RF magnetron sputtering onto Si(100, p-type) substrates, Normax glass slides (76 × 26 × 1 mm^3^), and pure aluminum pellets (0.7 cm diameter). Prior to deposition, all substrates were sequentially ultrasonically cleaned in deionized water, acetone, and ethanol for 10 min each, followed by hot-air drying and a final isopropanol rinse. Aluminum substrates were used for electrochemical characterization, while Si and glass substrates were employed for morphological and structural analyses, respectively.

Deposition was carried out in a laboratory vacuum chamber with a base pressure below 10^−2^ Pa and a working pressure of 0.7 Pa using Ar as the working gas. LiCoO_2_ and Ni targets (both ∼99% purity, Testbourne Ltd, 200 cm^2^ surface area) were co-sputtered for 3 h. The LiCoO_2_ target was powered by an RF generator (Hüttinger PFG 600) operating at a constant power of 200 W, while the Ni target was driven by a pulsed DC power supply (ENI RPG-50; 200 kHz frequency, 1536 ns pulse width).

To obtain films with different Ni incorporation profiles, the Ni target current was either maintained at a constant value of 0.25 A (uniform incorporation) or progressively ramped from 0.20 to 0.40 A in increments of 0.01 A every 9 min (gradient incorporation). A substrate-to-target distance of 70 mm and a substrate rotation speed of 7 rpm were maintained to ensure deposition uniformity. After deposition, the samples were immediately transferred to vacuum storage to minimize surface contamination and preserve coating integrity. Although the base pressure and working pressure were kept constant, minor variations in post-deposition ambient exposure time cannot be fully excluded and may contribute to differences in surface carbonate/hydroxyl content.

The deposition conditions for the LiNi_*x*_Co_1−*x*_O_2_ coatings, designated as LCO (pure LiCoO_2_), LNCO-0.25A (deposited with a constant Ni target current of 0.25 A), and LNCO-G (deposited with a linear Ni target current gradient from 0.20 to 0.40 A), are summarized in Table 1.

**Table 1:** Deposition conditions, thickness, and deposition rate of the three coatings

Coatings	LiCoO_2_ target power (W)	Ni target current (A)	Working pressure (Pa)	Thickness (nm)	Deposition rate (nm min^−1^)
LCO	200	—	0.7	415	2.31
LNCO-0.25A	200	0.25	0.7	723	4.01
LNCO-G	200	0.2 → 0.4	0.7	851	4.73

Although the film thickness differs between samples, the electrochemical comparison focuses on LNCO-0.25A and LNCO-G, which exhibit reasonably comparable thicknesses (∼723 *vs.* ∼851 nm, respectively) and deposition rates (4.01 and 4.73 nm min^−1^, respectively).

Current was normalized to the geometric electrode area to compare the areal electrochemical response of the coatings, reducing, but not eliminating, the influence of geometric loading on their comparison. Because these coatings also differ in total Ni content and phase composition, their area-normalized responses should be interpreted as reflecting the combined effects of composition, microstructure, and Ni distribution.

### 2.2. Sample characterization

The structural properties of the coatings were analyzed by X-ray diffraction (XRD), with grazing incidence (GIXRD) performed on a Philips X'Pert Pro diffractometer using Co Kα radiation (*λ* = 0.179 nm) at an incidence angle of 2°.

Vibrational properties were examined by Raman spectroscopy at room temperature using a Renishaw inVia™ confocal microscope with a 532 nm excitation wavelength over the range 200–1200 cm^−1^. Raman spectra were baseline-corrected and deconvoluted by fitting the broadened bands with a sum of Gaussian functions. The number and initial positions of the component peaks were estimated based on literature data, and the fitting procedure was iteratively refined until convergence. The quantity of the fits was assessed using the reduced *χ*^2^ parameter.

Morphological characterization was carried out using field-emission scanning electron microscopy (FESEM – Zeiss Merlin) operated at an accelerating voltage of 2 kV. Film thicknesses were determined from cross-sectional images. Elemental composition was analyzed by energy-dispersive X-ray spectroscopy (EDS) using an accelerating voltage of 10 kV and a beam current of 1 nA, including cross-sectional compositional profiling. To ensure statistical reliability, EDS measurements were averaged over three distinct regions, with identical analyzed areas. Cross-sectional EDS line scans were also acquired under identical instrumental conditions for all samples. EDS cannot detect hydrogen and does not distinguish among lattice oxygen, carbonate, hydroxide, or adsorbed water. C and O contents are therefore used as relative indicators of surface reactivity, not as absolute phase fractions.

Cross-sectional electron-transparent lamellae (<100 nm thick) of the LNCO-G sample were prepared using an FEI Helios NanoLab 450S focused ion beam system. High-angle annular dark-field scanning transmission electron microscopy (HAADF-STEM) and elemental mapping were conducted on a probe-corrected FEI Titan G2 80-200 ChemiSTEM microscope operating at 200 keV. HAADF images (2048 × 2048 pixels) were recorded with a 21 mrad convergence angle and a 4 µs pixel dwell time. Energy-dispersive X-ray spectroscopy (EDS) maps (1024 × 1024 pixels) were acquired using a Super-X EDS detector with a 5 µs dwell time and a 15 min accumulation period. Data processing was performed using Bruker Esprit software.

The chemical states and depth profiles were investigated by X-ray photoelectron spectroscopy (XPS) using a Thermo Scientific Escalab 250Xi system with non-monochromatic Al Kα radiation, under a base pressure below 1 × 10^−8^ Pa. High-resolution spectra were recorded at a pass energy of 40 eV with a step size of 0.1 eV, following monoatomic Ar^+^ ion sputtering (1 keV, 3 µA) to a depth of approximately 286 nm. Binding energies were calibrated using the C 1s peak at 285.0 eV with CasaXPS software.

Electrochemical performance was assessed using a Swagelok-type two-electrode cell (20 mm diameter) assembled in Ar-filled glovebox. The LiCoO_2_-based films served as the working electrode, while metallic lithium was used as both counter and reference electrode. The electrolyte consisted of 1 M of lithium perchlorate (LiClO_4_) dissolved in a 1 : 1 (v/v) mixture of ethylene carbonate (EC) and dimethyl carbonate (DEC). Measurements were carried out using GAMRY Interface 5000E potentiostat. Cyclic voltammetry (CV) was performed between 2.5 and 4.9 V *vs.* Li/Li^+^ at a scan rate of 0.5 mV s^−1^, while galvanostatic charge–discharge (GCD) measurements were conducted at a rate of 0.5C within a voltage window of 1.0–4.9 V.

The active mass (*m*) of each coating was determined using a geometric calculation (*m* = *ρ* × *A* × *t*), where *A* = 0.7 cm^2^ is the geometric active area exposed to the electrolyte, *t* is the cross-sectional film thickness measured by SEM (Table 1), and *ρ* = 5.05 g cm^−3^ is the theoretical density of LiCoO_2_. Although the theoretical crystallographic density of LiNi_*x*_Co_1−*x*_O_2_ decreases slightly with increasing nickel content (from 5.05 g cm^−3^ for *x* = 0 to 4.80 g cm^−3^ for *x* = 1), the maximum variation remains under 4.5%. Applying 5.05 g cm^−3^ across all samples provides a consistent reference baseline for active mass determination.

The practical discharge capacity corresponding to a nominal 1C rate was determined from CV recorded at low sweep rates. The absolute applied current (*I*) for each film was calculated individually based on its specific active mass, accounting for variations in thickness and composition to ensure equivalent current loads per unit mass across all tested electrodes.

To evaluate the interfacial stability and charge-transport kinetics of the coatings under aggressive operating conditions, electrochemical impedance spectroscopy (EIS) measurements were carried out after electrochemical cycling. The Nyquist spectra were fitted using a Randles-type equivalent circuit consisting of the series resistance (*R*_s_) in series with a parallel charge-transfer resistance/constant-phase-element branch (*R*_ct_‖CPE), followed by a Warburg diffusion element (*Z*_W_). The fitted parameters were used to compare ohmic, interfacial charge-transfer, and diffusion-related impedance among the coatings.

Both electrochemical techniques were intentionally performed over extended high-voltage ranges to probe the stability limits of the layered structure and to accentuate differences arising from Ni incorporation strategy. Although these voltage windows exceed those typically employed in commercial Li-ion batteries, they are commonly used in thin film cathode studies to evaluate lattice stability interface reactivity, and oxygen framework robustness at high states of charge.^21^

## 3. Results and discussion

### 3.1. Microstructural features

Fig. 1 shows the X-ray diffractograms of the LCO, LNCO-0.25A, and LNCO-G samples. The LCO film exhibits a weak and broadened reflection corresponding to the (006) plane at approximately 44.8° (rhombohedral-LiCoO_2_ – ICDD: 00-050-0653). The low intensity and significant peak broadening indicate that the LCO film is largely amorphous or poorly ordered, which is typical for as-deposited LiCoO_2_ thin films prepared by sputtering without post-deposition thermal treatment.^24^

**Fig. 1:**
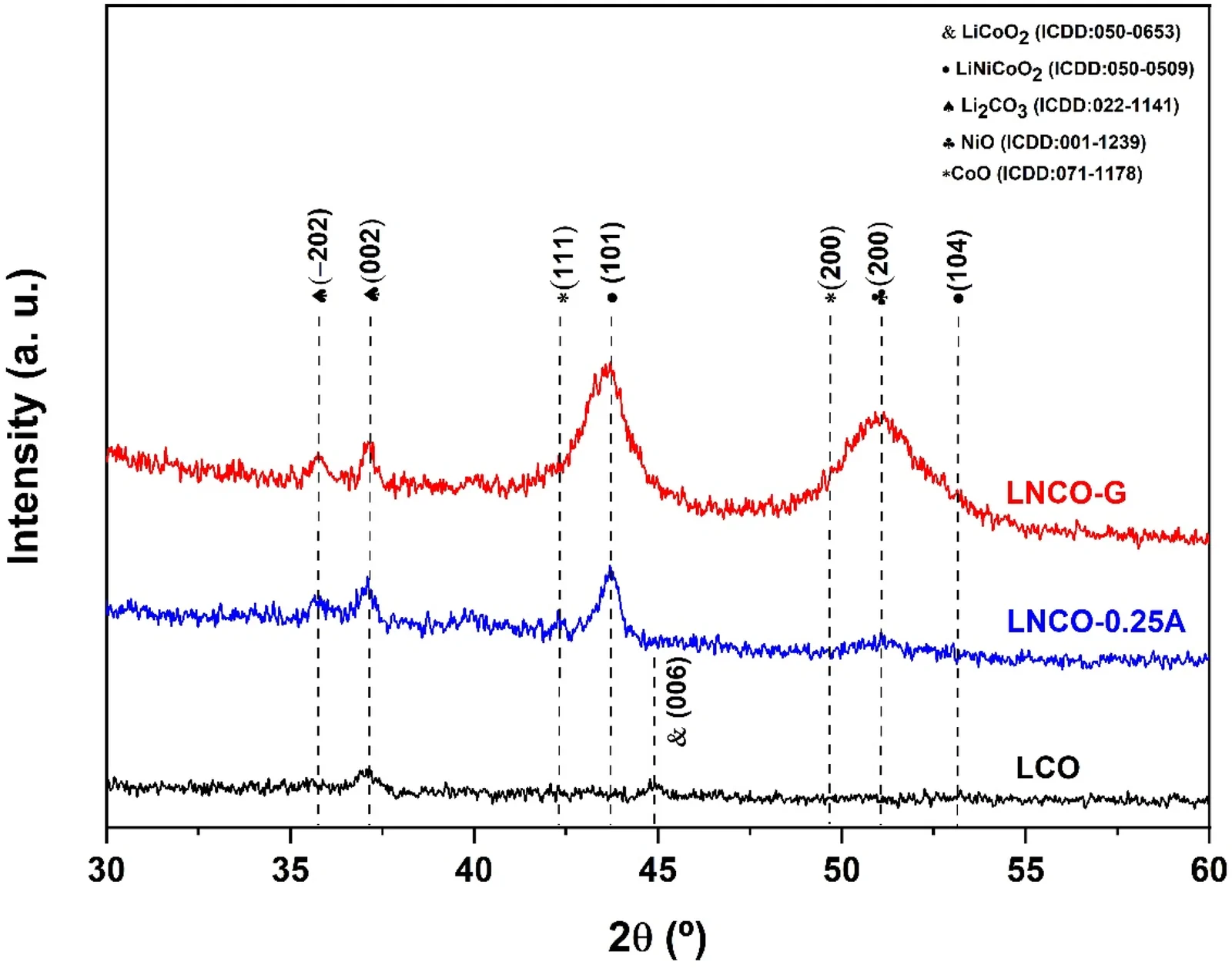
X-ray diffractograms of LCO, LNCO-0.25A, and LNCO-G.

In contrast, the LNCO samples display sharper and more intense peaks, indicating a higher degree of crystallinity compared to LCO. Notably, both Ni-incorporated films exhibit a preferred orientation with a dominant (101) reflection at approximately 43.9°. This peak can be assigned to a layered LiNi_*x*_Co_1−*x*_O_2_ phase (ICDD: 00-050-0509), which crystallizes in an α-NaFeO_2_-type structure with rhombohedral symmetry (space group *R*3̄*m*).^16^

The emergence of this preferential orientation suggests that Ni incorporation promotes the formation of a more ordered layered structure during film growth. However, the presence of Ni in the lattice may also introduce competing structural effects. Due to the similar ionic radii Ni^2+^ (0.69 Å) and Li^+^ (0.76 Å), Ni can partially occupy lithium sites, facilitating cation mixing and inducing local lattice distortions.^25,26^ These competing effects, enhanced crystallinity *versus* defect formation, indicate that the microstructure evolves through a balance between improved lattice ordering and the tendency toward cation disorder and secondary phase formation.

It should be emphasized that the XRD patterns are consistent with the presence of a layered LiNi_*x*_Co_1−*x*_O_2_-like phase, although the limited crystallinity of the as-deposited films prevents definitive space-group refinement. Because no post-deposition annealing was applied, long-range crystallographic ordering remains modest, and the *R*3̄*m* assignment is therefore made tentatively rather than conclusively. Quantitative Rietveld analysis was attempted but did not converge reliably, as the low crystallinity and overlapping reflections of the secondary phases preclude reliable structural refinement.

Several minor secondary phases are also detected. In the 35°–41° 2*θ* range, weak reflections corresponding to the (−202) and (002) planes of monoclinic Li_2_CO_3_ (ICDD: 00-022-1141) are observed. Given their low intensity, these carbonate-related peaks are most likely associated with exposure of the films to ambient air, as previously reported by Zhuang *et al.*^27^ The formation of Li_2_CO_3_ is commonly attributed to surface reactions of LiCoO_2_ with atmospheric CO_2_ and H_2_O and is frequently detected in both XRD and XPS.^24^

In addition, the formation of secondary transition metal oxides, such as NiO and CoO, is identified and appears to be influenced by the deposition conditions. In particular, the LNCO-G sample, prepared using a graded Ni incorporation strategy, exhibits a broad feature in the 48°–55° 2*θ* range. This feature can be interpreted as the superposition of multiple contributions, primarily associated with the (200) reflection of rock-salt NiO (fcc-NiO – ICDD: 00-001-1239) at ∼51°, overlapping with the (200) reflection of the CoO (fcc-CoO – ICDD: 01-071-1178) at ∼48°. A contribution from, the (104) reflection of the layered LiNi_*x*_Co_1−*x*_O_2_ phase may also be present within this region.

The relatively pronounced presence of NiO in the LNCO-G sample can be rationalized in terms of both thermodynamic and kinetic factors. Although NiO and CoO exhibit similar standard enthalpies of formation 
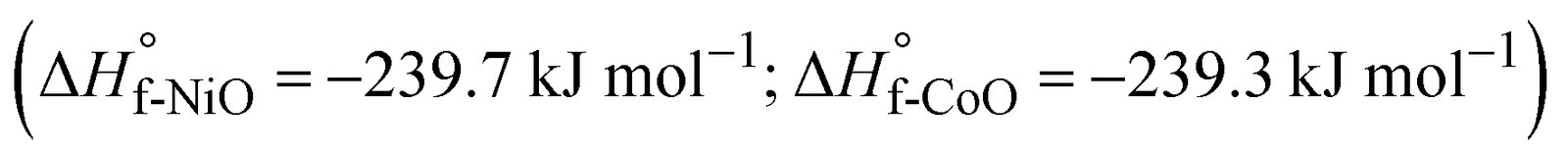
, the progressively increased Ni flux during gradient deposition is likely to favor the nucleation and growth of NiO-rich domains. While such secondary oxide phases are often considered undesirable, previous studies have shown that multiphase cathode systems can still exhibit favorable electrochemical performance and structural tolerance under demanding operating conditions.^28^ The possible contribution of these secondary phases to the electrochemical response is discussed in Section 3.5.

### 3.2. Raman spectroscopy

Fig. 2 displays the Raman spectra of the coatings deposited on glass substrates. All samples exhibit two characteristic vibrational modes: the E_g_ mode and the A_1g_ mode located at approximately 485 cm^−1^ and 589 cm^−1^, respectively. These modes are associated with the in-plane bending (E_g_) and out-of-plane stretching (A_1g_) vibrations of oxygen atoms in the layered LiCoO_2_.^24,29^ Specifically, the A_1g_ mode corresponds to the oxygen motion along the *c*-axis, while the E_g_ mode is related to in-plane vibrations perpendicular to this axis within the LiCoO_2_ layers.^30^ The presence of these characteristic bands in the Ni-incorporated samples indicates preservation of the layered structure and its underlying crystallographic symmetry, in agreement with previous reports.^31^ Differences in peak shape and intensity provide insight into structural ordering. In particular, sharper and more intense E_g_ and A_1g_ peaks are generally indicative of improved local structural order and reduced local disorder, although their evolution must be interpreted with care in the presence of secondary phases.^32^ The LNCO-0.25A sample exhibits an additional intense band at approximately 557 cm^−1^, which suggests the presence of secondary phases. Furthermore, all spectra display a band at ∼1089 cm^−1^ assigned to the symmetric stretching of CO_3_^2−^ groups in Li_2_CO_3_.^24^ This feature appears most intense in LNCO-0.25A, indicating a higher carbonate contribution.

**Fig. 2:**
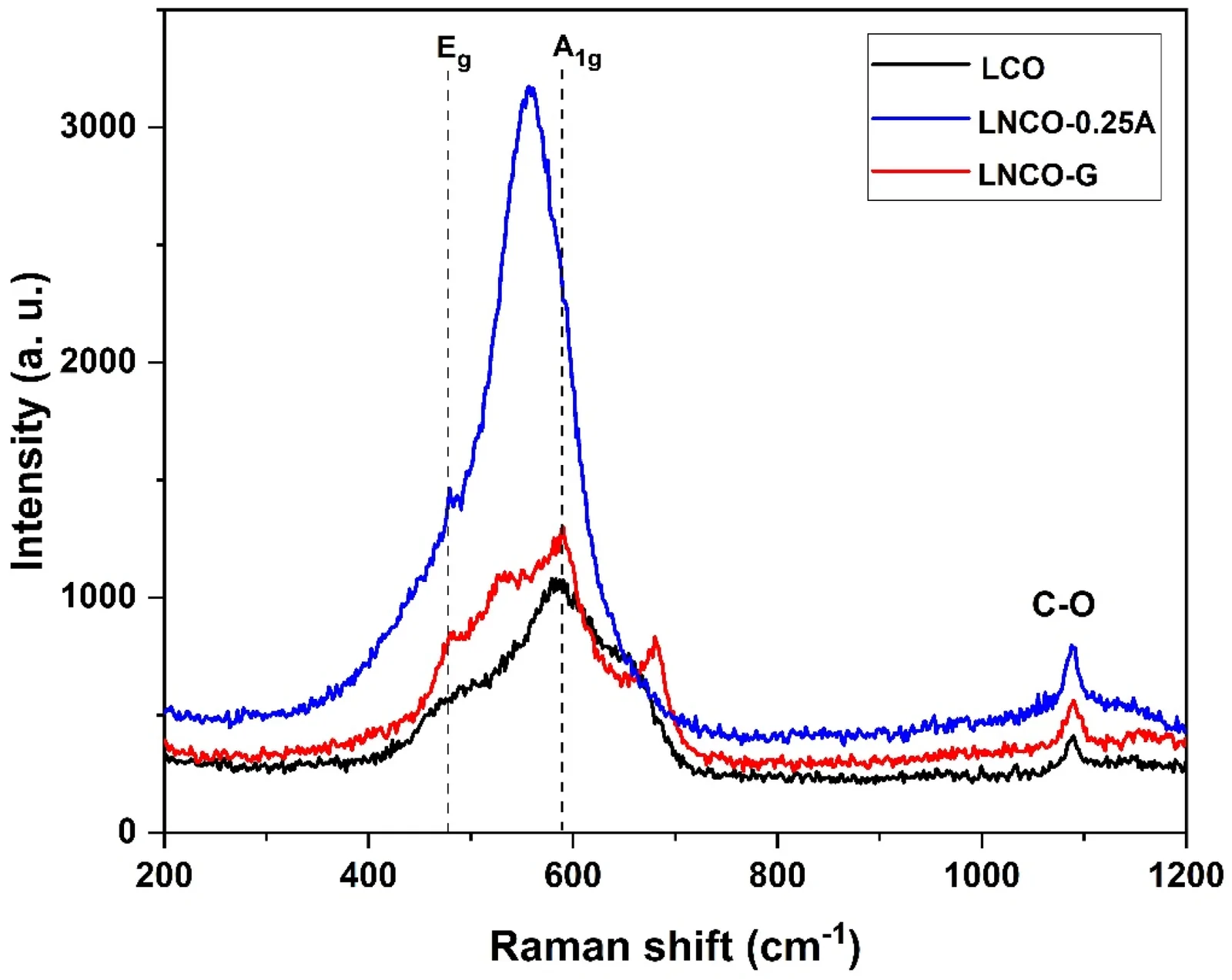
Room temperature Raman spectra of LCO, LNCO-0.25A, and LNCO-G coatings.

To gain further insight, each spectrum was deconvoluted. For the LCO sample (Fig. S1a), three bands are identified at 489, 589, and 660 cm^−1^. The first two correspond to the E_g_ and A_1g_ modes, while the band at 660 cm^−1^ is attributed to Co–O bonding vibrations.^33^

Fig. S1b reveals the deconvolution of the Raman curve for the LNCO-0.25A sample. The E_g_ (489 cm^−1^) and A_1g_ (589 cm^−1^) modes remain detectable; however, the A_1g_ peak broadens significantly (FWHM increases from 89.3 cm^−1^ in LCO to 154.6 cm^−1^), and the *I*(A_1g_)/*I*(E_g_) intensity ratio decreases from 1.9 to 1.4. This trend suggests increased local structural disorder and possible cation mixing. Such behavior is consistent with previous studies reporting reduced mode symmetry upon uniform Ni incorporation.^31^ Additional bands at approximately 447, 520, and 557 cm^−1^ are observed and can be attributed to Ni(OH)_2_, Co(OH)_2_, and NiOOH-related species, respectively,^34–36^ indicating a more chemically heterogeneous surface with secondary-phase intensity ratios relative to A_1g_ (*I*_phase_/*I*(A_1g_)) of 0.8, 1.8, and 2.6, respectively.

In contrast, the LNCO-G sample (Fig. S1c) exhibits narrower E_g_ and A_1g_ bands (FWHM of 54.9 cm^−1^ and 86.9 cm^−1^, respectively) and a higher *I*(A_1g_)/*I*(E_g_) ratio of 2.1. Both modes shift toward lower wavenumbers (485 cm^−1^ for E_g_ and 585 cm^−1^ for A_1g_), which may be associated with lattice expansion induced by gradient Ni incorporation.^30^ The lower peak widths reflect fewer surface defects available for atmospheric chemisorption, consistent with the lower hydroxyl/carbonate contribution observed by XPS, as will be shown in Section 3.4. Secondary phases in LNCO-G appear at 443 cm^−1^ corresponding to Ni(OH)_2_,^36^ 529 cm^−1^ attributed to NiO,^37^ and 675 cm^−1^ associated with CoO,^38^ with substantially lower *I*_phase_/*I*(A_1g_) ratios (0.2, 0.5, and 0.5, respectively). All fitted peak positions, FWHM values, and relative intensity ratios are compiled in Table S1. Although Raman spectroscopy provides qualitative information on local bonding rather than direct measurements of bulk crystallinity, these semi-quantitative parameters reflect the improved structural ordering of the LNCO-G sample relative to LNCO-0.25A, supporting the XRD findings.

### 3.3. Morphology and compositional analysis

To evaluate the impact of Ni incorporation on film morphology and homogeneity, scanning electron microscopy (SEM) analysis was performed, as shown in Fig. 3. The surface morphology of all samples is characterized by a distinct granular structure (Fig. 3a, d, and g). This morphology is consistent with previous observations^24^ and can be primarily attributed to the formation of Li_2_CO_3_ upon exposure to ambient atmosphere.

**Fig. 3:**
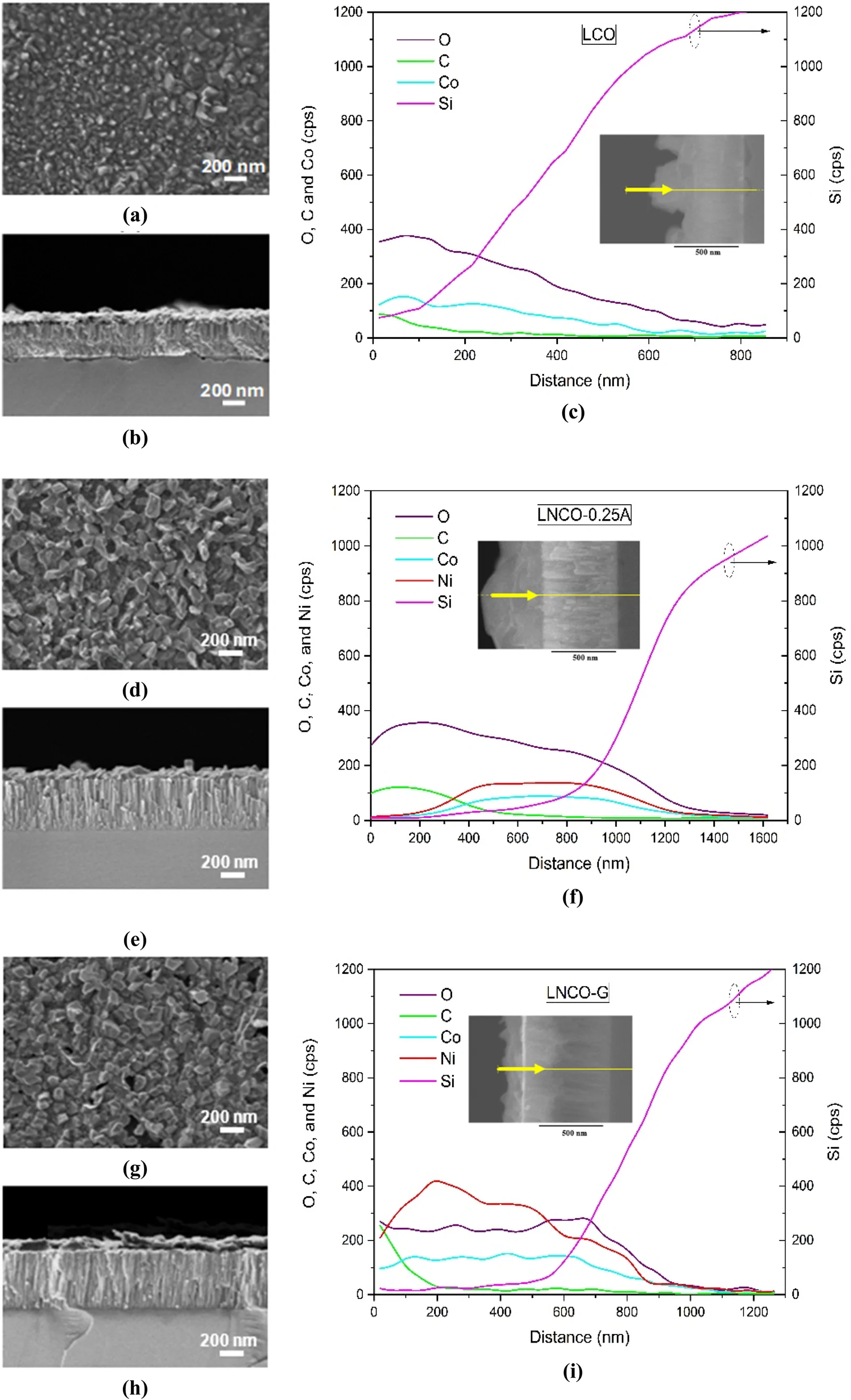
Surface morphology, cross-sectional SEM images, and depth-resolved EDS (10 keV) concentration profiles for (a–c) LCO, (d–f) LNCO-0.25A, and (g–i) LNCO-G thin films, respectively. Arrows indicate the direction of the profiling.

Notably, the LCO sample exhibits a relatively fine-grained surface morphology, whereas the Ni-incorporated samples display a coarser, more irregular “rocky-like” texture. These morphological differences suggest that Ni incorporation influences surface growth dynamics and post-deposition surface reactivity. Cross-sectional images (Fig. 3b, e, and h) reveal a columnar growth structure for all samples, consistent with the transition zone described in the Thornton structure zone model (SZM) for sputtered thin films.^24^

The average elemental composition obtained from energy-dispersive X-ray spectroscopy (EDS) analysis is summarized in Table 2. The results reveal substantial differences in the surface and bulk chemistry of the films as a function of the Ni incorporation strategy. The uniformly Ni-incorporated sample (LNCO-0.25A) exhibits the highest carbon content (26.8 at%) together with an elevated oxygen concentration (61.7 at%), indicating a greater tendency toward the formation of carbonate- and hydroxide-containing surface species. In contrast, LNCO-G presents lower carbon (21.1 at%) and oxygen (47.4 at%) contents despite containing a substantially higher Ni concentration (26.3 at%). This behavior suggests that the gradient Ni incorporation modifies the chemical evolution of the film surface and limits the accumulation of oxygen-rich reaction products. These compositional trends are consistent with the XPS results, which indicate a reduced contribution of hydroxyl and carbonate species in LNCO-G compared with LNCO-0.25A.

**Table 2:** Average elemental composition (at%) of the investigated coatings obtained from EDS analysis

Element (at%)	LCO	LNCO-0.25A	LNCO-G
C	24.4	26.8	21.1
O	68.2	61.7	47.4
Co	7.4	4.9	5.2
Ni	—	6.6	26.3
*x* = Ni/(Ni + Co)	—	0.57	0.83

To establish the transition-metal stoichiometry defined by the general formula LiNi_*x*_Co_1−*x*_O_2_, the atomic fraction *x* = Ni/(Ni + Co) was calculated from quantitative EDS analysis (Table 2). For the uniform LNCO-0.25A coating, the average transition-metal ratio yields *x* ≈ 0.57, corresponding to a homogeneous LiNi_0.57_Co_0.43_O_2_ composition. For the gradient film (LNCO-G), area-averaged EDS yields an integrated bulk stoichiometry of *x̄* ≈ 0.83. However, assigning a single static *x* value to LNCO-G does not reflect its physical structure. Because the film was deposited under a continuous current gradient, *x* varies as a function of depth (*x*(*z*)), as detailed in the subsequent cross-sectional STEM–EDS analysis (Fig. 4) where *x* increases continuously along the growth direction from the substrate interface toward the outer surface.

**Fig. 4:**
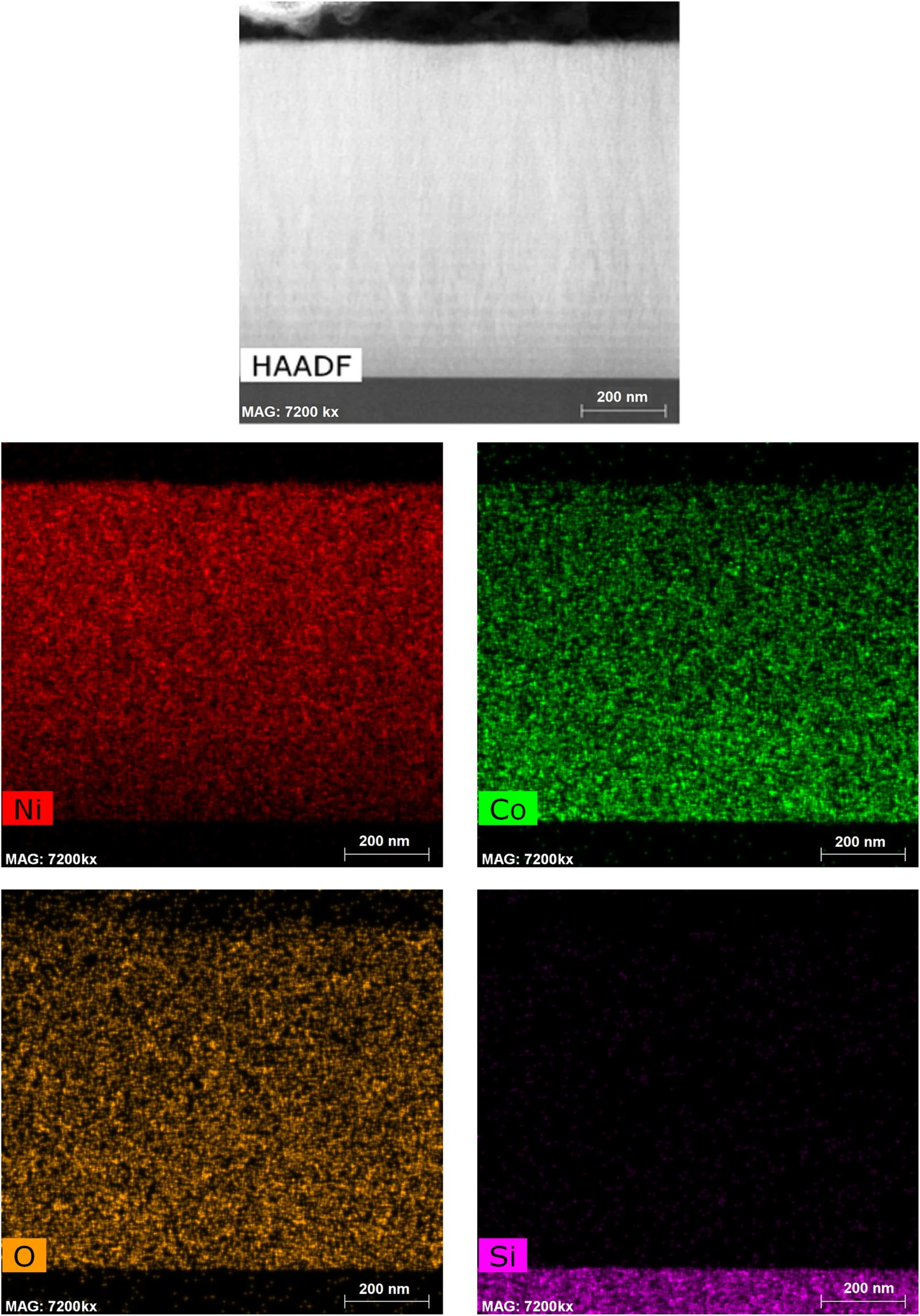
Correlation HAADF-STEM image of LNCO-G and EDS mapping of Ni, Co, O, and Si chemical elements.

To further investigate compositional variations within the coatings, depth-resolved EDS profiling was performed, as presented in Fig. 3. As noted in Section 2.2, the EDS trends are relative; the corresponding chemical states are identified by XPS.

For the LCO sample (Fig. 3c), the composition is dominated by Co and O, consistent with the expected stoichiometry. The relatively high oxygen signal suggests an oxidized surface state, which is consistent with the deposition conditions and subsequent air exposure. In the case of LNCO-0.25A (Fig. 3f), the EDS profile indicates a relatively uniform distribution of Ni throughout the film thickness, confirming homogeneous Ni incorporation under constant current conditions. The Co and O distributions remain comparable to those observed in the LCO sample. In contrast, the LNCO-G sample (Fig. 3i) exhibits a clear compositional gradient, with a higher Ni concentration near the film surface that gradually decreases toward the substrate. This depth-dependent distribution is consistent with the programmed variation of the Ni target current during deposition.

An additional feature observed in all samples is the presence of a carbon-rich surface layer, particularly pronounced in LNCO-0.25A. As shown in Fig. 3f, this surface region contains elevated C and O signals, consistent with the formation of Li_2_CO_3_, in agreement with the XRD and Raman analyses discussed earlier. These observations highlight the influence of both deposition conditions and post-deposition surface reactions on the chemical composition and microstructure of the films.

The observed differences in surface reactivity between uniform and gradient films may be partially rooted in microstructural and compositional heterogeneity. The denser columnar morphology of LNCO-G (Fig. 3h) implies a reduced density of grain boundaries and surface defects that typically serve as preferential adsorption sites for atmospheric H_2_O and CO_2_. Furthermore, the Ni-rich surface layer revealed by EDS profiling (Fig. 3i) suggests that the outermost surface is thermodynamically poised to form a NiO-dominated passivation layer upon ambient exposure. The formation of such a compact oxide overlayer may present a kinetic barrier to further hydration,^39^ consistent with the reduced hydroxyl contribution observed in Raman and subsequent XPS analyses.

Cross-sectional high-angle annular dark-field scanning transmission electron microscopy (HAADF-STEM) was used to examine the through-thickness structure of the LNCO-G film (Fig. 4). The image reveals atomic-number-dependent contrast (*Z*-contrast) variations along the growth direction. Subtle periodic modulations in the lower part of the coating are attributed to the rotation of the substrate holder between the LiCoO_2_ and Ni sputtering targets. During exposure to the LiCoO_2_ target, the deposited region is relatively Co–O-rich and therefore exhibits darker contrast, whereas exposure to the Ni target produces a Ni-enriched region with brighter contrast. As deposition proceeds and the growth conditions become more stable, the periodic contrast modulation becomes less pronounced, indicating a reduction in the amplitude of the local compositional fluctuations. This observation could be interpreted as a progressive homogenization of the local sub-layering within the overall columnar coating.

Elemental mapping by EDS confirms the spatial distribution of the constituent elements above the Si substrate. The Ni signal intensity increases progressively from the substrate interface toward the outer surface of the film, whereas Co and O remain broadly distributed throughout the coating thickness, with a modest relative decrease near the top region. Although the *Z*-contrast variations in the HAADF-STEM image capture the initial sub-layering induced by target alternation, the corresponding EDS maps depict the resulting through-thickness composition as a continuous macroscopic Ni gradient.

### 3.4. Surface chemistry and depth profiling

X-ray photoelectron spectroscopy (XPS) was employed to investigate the chemical states and depth-dependent surface chemistry of the coatings. Fig. 5 presents the survey spectra of the LCO, LNCO-0.25A, and LNCO-G samples. Photoemission signals corresponding to Co 2p, O 1s, C 1s, and Li 1s are observed in all samples, while Ni 2p is detected only in the Ni-incorporated coatings.

**Fig. 5:**
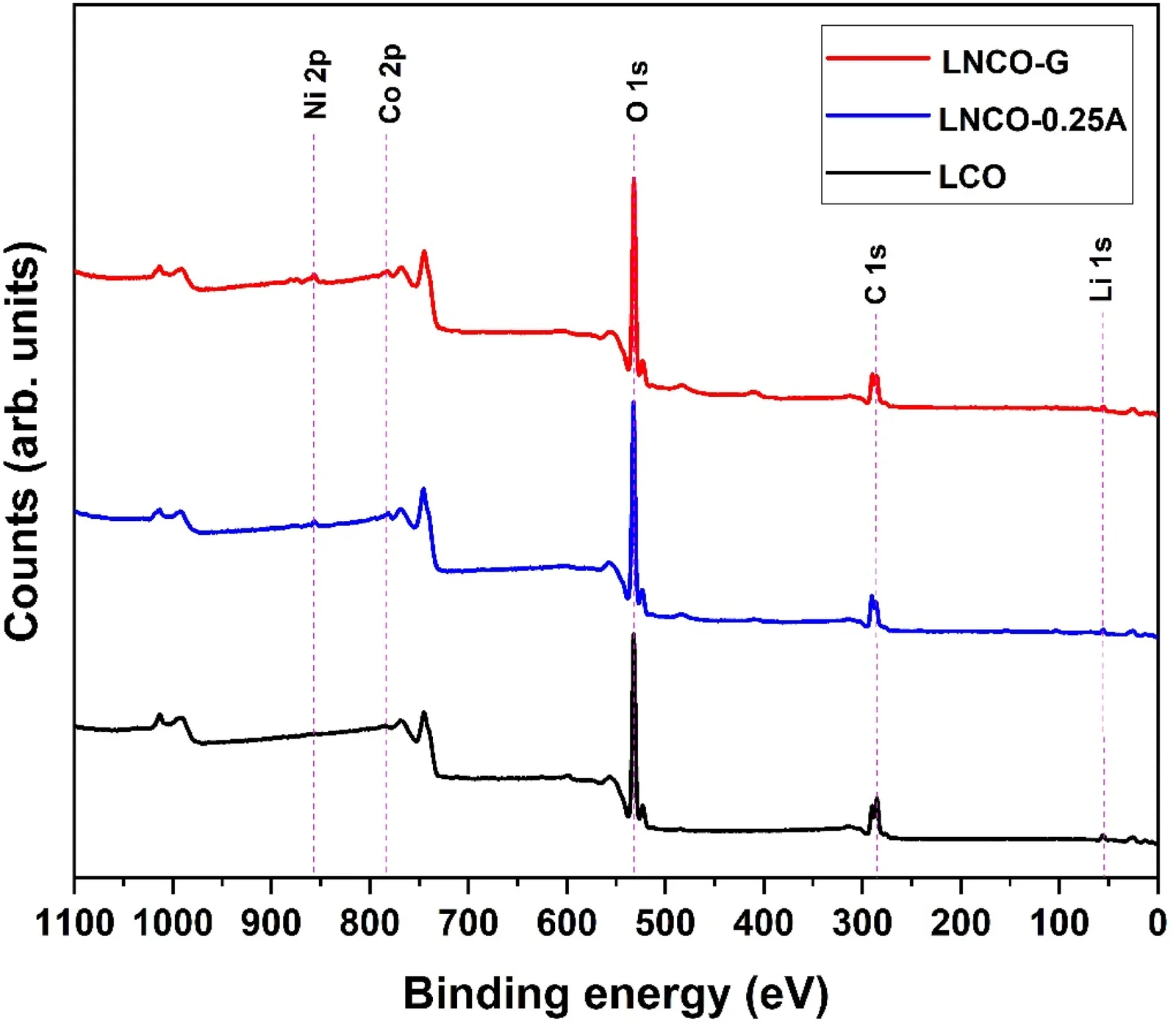
XPS survey spectra of LCO, LNCO-0.25A and LNCO-G samples.

The C 1s core-level spectra of the three coatings, obtained after Ar^+^ ion etching, are shown in Fig. 6. In all samples, a peak centered at ∼290.1 eV is observed and assigned to carbonate species (CO_3_^2−^), primarily Li_2_CO_3_, which is commonly reported in LiCoO_2_-based coatings exposed to ambient conditions.^40^ The persistence of this signal after ion etching suggests that carbonate species are not confined exclusively to the outermost surface and may extend into the near-surface region. In addition to the carbonate contribution, LNCO-0.25A exhibits components at ∼286.5 eV (C–OH), indicating a more chemically complex surface (Fig. 6b). The presence of these oxygen-containing carbon species suggests a greater susceptibility of this coating to reaction with atmospheric H_2_O and CO_2_, in agreement with previous reports.^41,42^

**Fig. 6:**
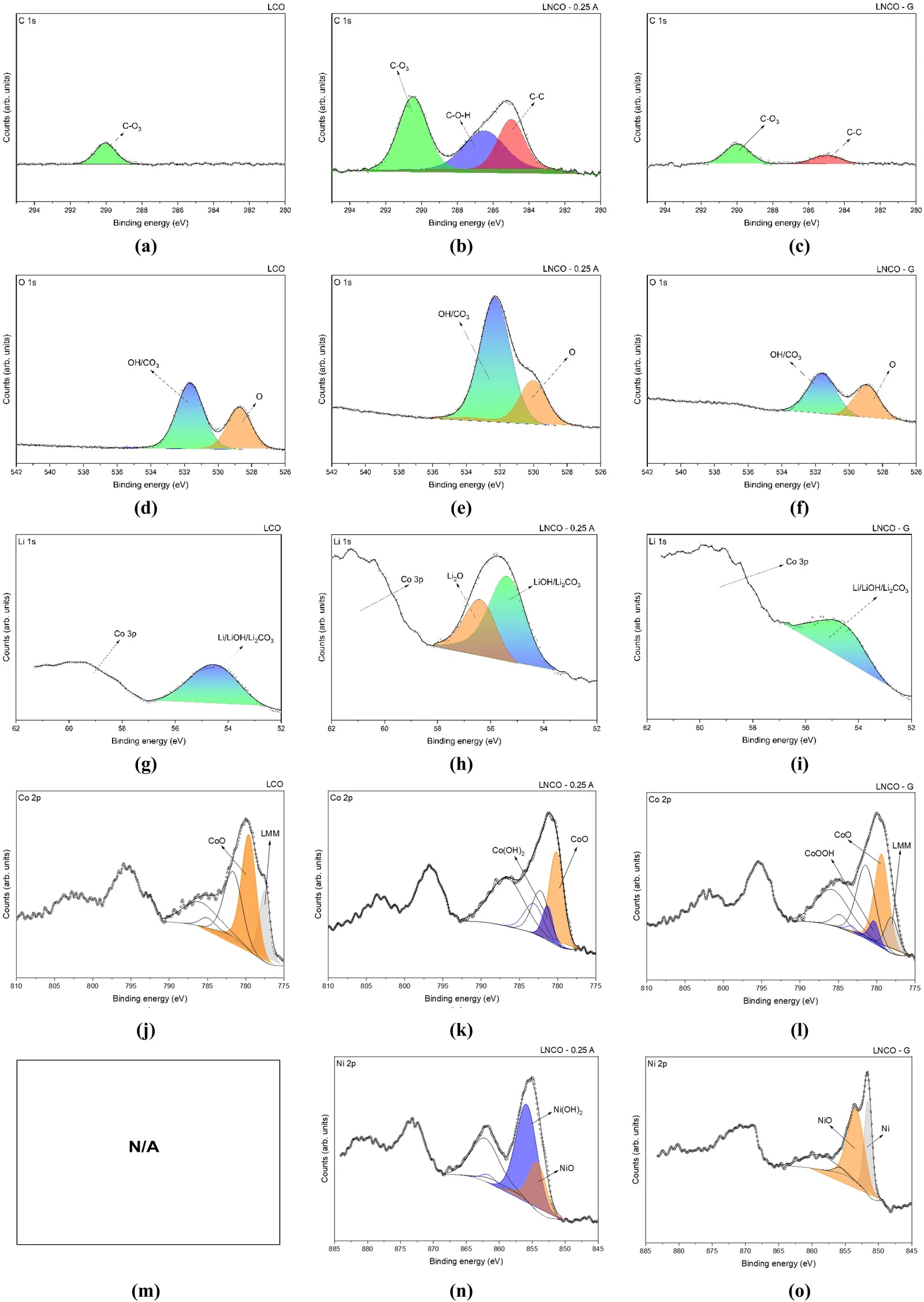
Core level XPS spectra of LCO, LNCO-0.25A, and LNCO-G obtained at an etching depth of 286 nm: (a–c) for C 1s, (d–f) for O 1s, (g–i) for Li 1s, (j–l) for Co 2p, and (m–o) for Ni 2p spectra, respectively.

Similar to the other samples, the C 1s spectrum of LNCO-G is dominated by the carbonate-related peak at ∼290.1 eV (Fig. 6c). However, compared with LNCO-0.25A, the spectrum exhibits less pronounced C–OH contributions, indicating a lower degree of surface hydration and chemical heterogeneity. After etching, the carbonate signal remains detectable, suggesting that carbonate-containing species are distributed throughout the near-surface region rather than being restricted to the outermost surface. Given the sensitivity of surface carbon species to environmental exposure, and despite the precautions mentioned in Section 2.1, the possibility that uncontrolled adsorption processes contributed to the observed spectral differences cannot be entirely excluded.

These observations are consistent with the Raman and EDS results. The stronger carbonate-related Raman band and higher carbon content observed for LNCO-0.25A indicate a greater tendency toward surface carbonation and hydration, whereas LNCO-G exhibits lower carbon enrichment despite its substantially higher Ni content. Taken together, these results suggest that gradient Ni incorporation does not suppress carbonate formation entirely but reduces the accumulation of hydroxylated and oxygen-containing carbon species. Consequently, LNCO-G develops a less hydrated and chemically more stable near-surface environment than LNCO-0.25A, which is expected to contribute positively to interfacial stability and electrochemical performance.

The O 1s spectra, which is shown in Fig. 6, exhibit two main contributions centered at ∼528.9 eV and ∼531.8 eV. The lower binding energy peak is assigned to lattice oxygen (O^2−^) in metal–oxygen bonds, whereas the higher binding energy component corresponds to overlapping contributions from hydroxyl (–OH) and carbonate (CO_3_^2−^) species.^43^

After Ar^+^ ion etching, LNCO-0.25A retains a relatively strong OH/CO_3_ contribution (Fig. 6e), indicating the persistence of surface-derived species and suggesting a higher degree of surface reactivity. In contrast, LNCO-G exhibits a markedly reduced OH/CO_3_ contribution (Fig. 6f), consistent with a less hydrated near-surface environment. These observations indicate that gradient Ni incorporation limits the accumulation of hydroxylated and carbonate-containing surface species.

The O 1s results are consistent with both the Raman and EDS analyses. LNCO-0.25A exhibits the strongest carbonate-related Raman band together with the highest carbon and oxygen contents measured by EDS, indicating a greater tendency toward hydration and carbonation. Conversely, LNCO-G shows lower carbon enrichment and reduced hydroxyl/carbonate contributions despite its substantially higher Ni content. This agreement among Raman, EDS, and XPS analyses indicates that the Ni incorporation strategy strongly influences the formation and distribution of surface reaction products.

Taken together, these findings suggest that gradient Ni incorporation does not completely suppress surface carbonation but reduces the accumulation of highly reactive hydroxylated species, leading to a chemically more stable interface. The LCO sample exhibits intermediate behavior (Fig. 6d), with contributions from both lattice oxygen and surface-derived species, consistent with a partially hydrated surface.

The formation of Li_2_CO_3_ and hydroxyl-containing species is not exclusively a consequence of post-deposition atmospheric exposure. Recent studies on Ni-containing layered oxides have shown that the spontaneous reduction of higher-valence Ni species can generate reactive oxygen-containing surface environments that subsequently react with H_2_O and CO_2_, promoting the formation of LiOH and Li_2_CO_3_.^44,45^ Consequently, the extent of surface carbonation and hydration is closely linked to the local Ni chemistry and surface stability of the oxide framework.^41^ Consistent with this mechanism, LNCO-0.25A exhibits stronger hydroxyl and carbonate signatures than LNCO-G, suggesting that the Ni incorporation strategy influences the evolution of these surface reaction products rather than merely their exposure history.^46^

Fig. 6 presents the Li 1s core-level spectra of the investigated coatings following Ar^+^ ion etching. The etched LCO and LNCO-G samples exhibit a large peak centered at ∼54.98 eV (Fig. 6g and i). According to Moulder *et al.*,^47^ this binding energy corresponds to overlapping contributions from LiOH (54.8–55.3 eV), Li_2_CO_3_ (54.9–55.4 eV), and metallic Li (54.5–55.0 eV). In contrast, LNCO-0.25A displays a broader Li 1s signal that can be deconvoluted into two components centered at ∼55.35 eV and ∼56.37 eV (Fig. 6h). The former is consistent with LiOH/Li_2_CO_3_ species, whereas the latter can be assigned to Li_2_O.^47^ The broader peak distribution observed in LNCO-0.25A suggests a more complex lithium chemical environment, consistent with the higher degree of surface carbonation and hydroxylation inferred from the C 1s and O 1s spectra.

Furthermore, Ar^+^ ion etching reveals the Co 3p core-level signal in all samples, indicating that cobalt is predominantly located beneath the surface layer rather than at the outermost surface. This observation is consistent with the EDS depth profiles (Fig. 3) and supports the existence of a surface region enriched in lithium-derived reaction products.

The Co 2p and Ni 2p core-level spectra become more clearly resolved after Ar^+^ ion etching, reflecting the removal of the surface reaction layer and enabling a more representative analysis of the underlying coating chemistry. Fig. 6j–l present the Co 2p spectra of the investigated coatings. All samples exhibit two main peaks at ∼779.6 eV and ∼794.8 eV, corresponding to the Co 2p_3/2_ and Co 2p_1/2_ spin–orbit components, respectively, with a binding energy separation of ∼15.2 eV, in agreement with reported values.^47^

For the LCO sample, the Co 2p_3/2_ region shows a dominant contribution centered near ∼779.6 eV, consistent with CoO-like environments, accompanied by broader features at higher binding energies associated with plasmon loss structures (Fig. 6j). According to Biesinger *et al.*,^48^ these features can be distinguished into surface plasmons (∼3 eV above the main peak) and bulk plasmons (∼5 eV above the main peak). An additional peak at ∼777.5 eV is attributed to the Co L_3_M_2,3_M_4,5_ (^1^p) Auger transition.^47^

Both Ni-incorporated samples exhibit Co 2p components consistent with CoO-like environments; however, notable differences are observed in the higher binding energy contributions. In LNCO-0.25A, a component at ∼781.3 eV together with its associated plasmon feature is consistent with Co^2+^ species in hydroxide-rich environments (Fig. 6k),^48^ suggesting a greater degree of surface hydration. In contrast, LNCO-G exhibits a feature near ∼780.3 eV that is consistent with Co^3+^-like environments (Fig. 6l), such as CoOOH,^47^ indicating a different local chemical environment.

These observations support the conclusion that the Ni incorporation strategy influences the chemical state of cobalt and the extent of surface hydration. In particular, the reduced intensity of hydroxyl-related contributions observed in the O 1s spectra of LNCO-G is consistent with the lower abundance of Co hydroxide species inferred from the Co 2p analysis. This behavior further supports the view that gradient Ni incorporation suppresses the accumulation of highly reactive hydroxylated surface species, leading to a less hydrated and chemically more stable near-surface environment than that observed for LNCO-0.25A.

Fig. 6m–o present the Ni 2p core-level spectra. Pristine LCO (Fig. 6m) exhibits no nickel signal (labeled N/A), whereas Fig. 6n and o display the spectra for the Ni-incorporated samples (LNCO-0.25A and LNCO-G). For LNCO-0.25A, the main Ni 2p_3/2_ peak is centered at ∼855.8 eV and is assigned to Ni(OH)_2_, as evidenced by its characteristic satellite structure (Fig. 6n).^49^ This observation indicates significant surface hydroxylation, consistent with the hydrated surface chemistry inferred from the C 1s and O 1s analyses. A secondary contribution at ∼853.8 eV can be attributed to NiO,^50^ indicating the coexistence of hydroxide and oxide species within the coating. These results suggest that uniform Ni incorporation does not effectively suppress post-deposition oxidation and hydration processes.

In contrast, LNCO-G exhibits a markedly different spectral profile (Fig. 6o). The Ni 2p_3/2_ region is dominated by a contribution associated with NiO, together with a weaker component near ∼854.6 eV that may be attributed to partially oxidized or metallic Ni species.^47^ These features are accompanied by satellite structures in the 855–865 eV range, characteristic of Ni^2+^-containing environments.^48–51^ The energy separation of ∼15.5 eV between the main peak and satellite is consistent with reported values for partially oxidized nickel species.^48,51^

The coexistence of Ni^0^ and Ni^2+^ in LNCO-G suggests that a fraction of the incorporated Ni remains only partially oxidized, possibly due to local variations in oxygen availability during sputtering and/or incomplete oxidation during subsequent air exposure. Compared with LNCO-0.25A, the reduced intensity of Ni(OH)_2_-related features indicates a lower degree of surface hydroxylation and a less hydrated local chemical environment.

Overall, the Ni 2p analysis demonstrates that the Ni incorporation strategy strongly influences the chemical state of Ni within the coatings. Whereas LNCO-0.25A contains substantial hydroxide-related contributions, LNCO-G is characterized predominantly by oxide and partially reduced Ni species. The predominance of NiO-related contributions in LNCO-G is particularly noteworthy because NiO-like environments are frequently associated with surface reconstruction phenomena in Ni-containing layered oxides. Previous study has shown that NiO-rich surface regions can arise from local oxygen instabilities and may act as chemically distinct interfacial layers.^52^ Although the present work does not resolve the atomic structure of these regions, the enhanced NiO contribution observed in LNCO-G suggests that gradient Ni incorporation modifies the surface reconstruction pathway and associated reaction chemistry.

These differences are expected to influence interfacial reactions, surface charge characteristics, and ultimately the electrochemical response of the films.^53^

More generally, XPS analysis reveals that all coatings undergo surface carbonation upon exposure to ambient air, leading to the formation of Li_2_CO_3_. This process is thermodynamically favored because the Gibbs free energy of formation of Li_2_CO_3_
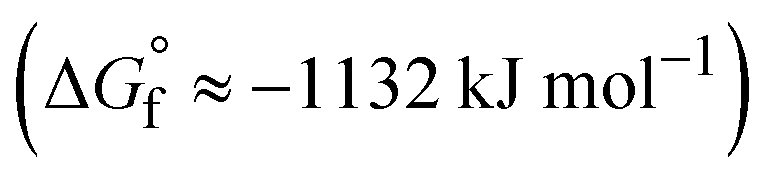
 is considerably lower than that of the corresponding hydroxides (LiOH, Ni(OH)_2_, Co(OH)_2_, and CoOOH) and oxides (NiO and CoO) 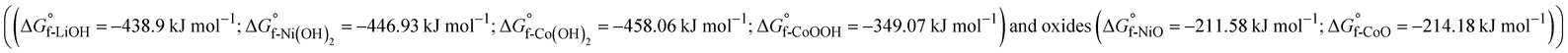.^54^ Consequently, the surface chemistry is dominated by carbonate formation, followed by varying degrees of hydration and oxidation.

Within this framework, the gradient Ni incorporation strategy appears to moderate these reactions, resulting in a lower abundance of hydroxylated species and a more controlled distribution of chemical states in both Li and Ni. The carbonate-rich surface layer detected in all samples is a common air-exposure product and is generally detrimental to interfacial transport. In LNCO-G, however, its amount and associated hydroxyl content are lower, which is consistent with a less reactive near-surface chemistry rather than a beneficial passivating role of Li_2_CO_3_ itself. Together with the Raman and EDS results, these findings indicate that gradient Ni incorporation promotes a less reactive surface environment than uniform Ni incorporation. All fitted XPS parameters are summarized in Table S2.

The distinct surface chemistries observed for LNCO-0.25A and LNCO-G can be rationalized by considering the coupled evolution of structural ordering, Ni chemistry, and surface reactivity. In the uniformly Ni-incorporated film, the homogeneous distribution of Ni appears to promote a chemically heterogeneous surface characterized by a greater abundance of hydroxylated and carbonate-containing species, as evidenced by Raman, EDS, and XPS analyses. Previous studies on Ni-containing layered oxides have shown that surface carbonation and hydroxylation are closely associated with the local Ni oxidation state and the interaction of reactive surface oxygen species with atmospheric H_2_O and CO_2_,^52^ ultimately leading to the formation of LiOH and Li_2_CO_3_-rich surface layers. These species are known to increase interfacial reactivity and contribute to charge-transfer limitations.^55,56^

In contrast, the compositional gradient established in LNCO-G introduces a progressive variation in local composition through the film thickness, accompanied by enhanced crystallinity and reduced structural disorder, as evidenced by XRD and Raman spectroscopy. The reduced Raman peak broadening and better-resolved vibrational features suggest a lower density of structurally reactive sites available for atmospheric chemisorption. Furthermore, the higher Ni concentration near the film surface and the predominance of NiO-related signatures in Raman and XPS analyses indicate the development of a Ni-rich oxide environment rather than a hydroxide-rich surface.

Several studies have shown that NiO-rich surface regions are chemically distinct from hydroxide-rich environments and may modify the pathway of surface reconstruction and interfacial reactions in Ni-containing layered oxides.^52,57^ Although the atomic-scale structure of these regions cannot be resolved in the present work, the reduced abundance of hydroxylated species observed in LNCO-G suggests that gradient Ni incorporation moderates the sequence of carbonation, hydration, and oxidation reactions occurring at the film surface.^58^

Taken together, these observations indicate that gradient Ni incorporation influences not only the bulk structure of the coating but also its surface chemical evolution. The resulting reduction in hydroxylation and surface reactivity is expected to improve interfacial stability and facilitate charge-transfer processes during electrochemical cycling, providing a mechanistic basis for the enhanced electrochemical response discussed in the following section.

### 3.5. Electrochemical analysis

The electrochemical behavior of the as-deposited LiCoO_2_-based films was evaluated by cyclic voltammetry (CV) and galvanostatic charge–discharge (GCD) measurements in a half-cell configuration using metallic lithium as the counter and reference electrode. The primary objective was to comparatively assess the electrochemical reversibility, polarization behavior, and structural stability of the coatings under high-voltage conditions (aggressive conditions).

Fig. 7a presents the first-cycle cyclic voltammograms of the three samples. The recorded CV curves were acquired at 0.5 mV s^−1^ to enable rapid comparative screening; the signal-to-noise ratio is limited by the low active mass of the sputtered films. The CV data are therefore interpreted comparatively rather than for quantitative kinetic analysis.

**Fig. 7:**
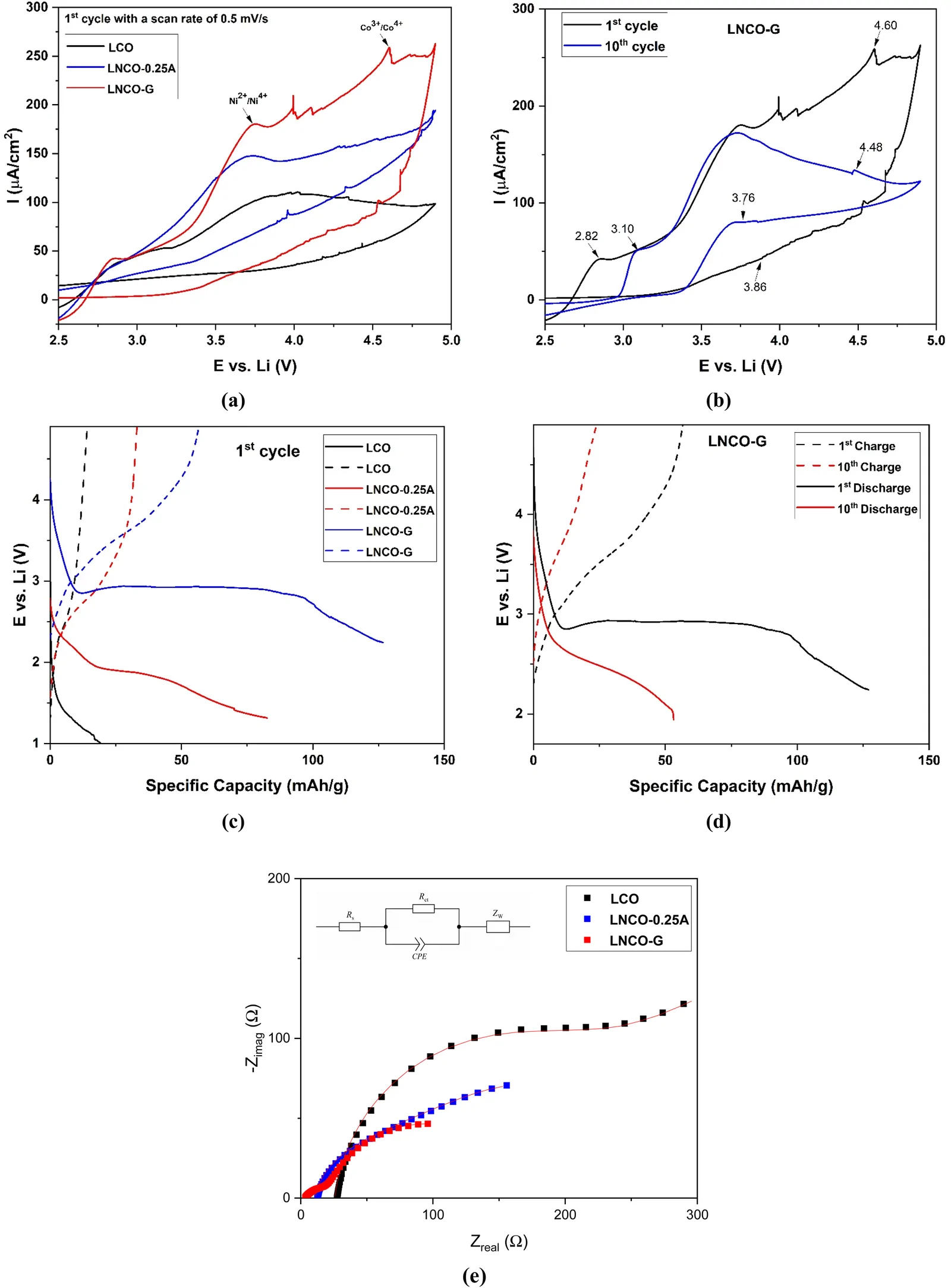
Electrochemical characterization of LCO, LNCO-0.25A, and LNCO-G thin films: (a) first-cycle cyclic voltammograms recorded at 0.5 mV s^−1^; (b) CV evolution of the LNCO-G film between the 1st and 10th cycles; (c) first-cycle galvanostatic charge–discharge curves measured at 0.5C; (d) charge–discharge curves of LNCO-G during the 1st and 10th cycles; and (e) post-cycling EIS Nyquist plots (symbols represent experimental data, and red solid lines represent the equivalent-circuit fits).

The LCO film exhibits relatively low current densities (∼50–70 µA cm^−2^) and broad, poorly defined redox features, indicating sluggish Li^+^ transport kinetics and significant electrochemical polarization. A diffuse anodic hump is observed in the 3.8–4.3 V region, which corresponds to high-voltage processes in LiCoO_2_ that are known to induce lattice strain, oxygen instability, and irreversible structural transformations.^59^

The LNCO-0.25A exhibits a moderate increase in current density compared to LCO, along with the emergence of additional anodic features around ∼4.5 V. However, both anodic and cathodic peaks remain broad and significantly separated, indicating pronounced polarization and limited electrochemical reversibility.

This behavior is consistent with the spectroscopic findings, which reveal a chemically heterogeneous surface enriched in hydroxylated and carbonate species, as evidenced by Raman and XPS analyses. In particular, the presence of Ni(OH)_2_/NiOOH-related environments suggests substantial surface hydration and chemical instability. Such surface species are known to destabilize the electrode–electrolyte interface, increase interfacial resistance, and hinder Li^+^ transport kinetics.

These observations contrast with previous strategies aimed at improving interfacial stability in layered oxide cathodes. For example, modest Al incorporation in high-Ni cathodes^56^ and surface engineering approaches, such as polymer coatings on LiCoO_2_,^60^ have been shown to enhance high-voltage performance by suppressing interfacial side reactions and stabilizing surface chemistry. In this context, the results obtained for LNCO-0.25A indicate that uniform Ni incorporation alone is insufficient to mitigate surface reactivity under the present processing conditions.

In contrast, the LNCO-G sample exhibits an enhanced electrochemical response, with peak current densities reaching ∼270 µA cm^−2^ and multiple well-defined redox features. A weak anodic feature is observed near ∼2.8 V, which can be attributed to minor surface- or defect-related processes. The main anodic peak centered at ∼3.7 V is associated with the Ni^2+^/Ni^4+^ redox couple,^61^ indicating active participation of Ni in the charge-transfer mechanism.

Additional anodic features in the ∼3.9–4.1 V range correspond to progressive lithium extraction accompanied by stepwise oxidation of Ni and Co species within the layered framework. At higher potentials, a feature above ∼4.6–4.7 V is attributed to the Co^3+^/Co^4+^ redox transition,^61^ while the broad signal extending beyond ∼4.6 V reflects deep delithiation processes under the extended voltage window employed.

The presence of multiple, partially overlapping anodic peaks suggests that redox reactions do not occur in a single uniform environment. Instead, these features can be interpreted as arising from electrochemical processes occurring in chemically and structurally non-equivalent regions along the Ni compositional gradient. This behavior is characteristic of compositionally graded and Ni-rich layered oxides, where spatial variations in local composition lead to distributed redox potentials.^60–62^

Fig. 7b compares the cyclic voltammograms of the LNCO-G sample recorded during the 1st and 10th cycles. A moderate decrease in current intensity is observed, accompanied by a slight reduction in the anodic–cathodic peak separation (Δ*E*_p_) of the main Ni/Co-based redox couple, from approximately 0.74 V in the first cycle to 0.72 V in the tenth cycle.

This evolution is also associated with gradual peak broadening, indicating increasing polarization and a progressive slowdown in charge-transfer kinetics upon cycling. Despite these changes, the overall voltammetric profile is largely preserved, suggesting that the same intercalation mechanism remains operative throughout cycling.

Similar behavior, including cathodic peak shifts and peak broadening, has been reported for Ni-rich layered oxides such as LiNiO_2_ and LiNi_0.8_Co_0.2_O_2_,^63^ where such changes are typically attributed to increased polarization and structural evolution under repeated lithium extraction and insertion.

Such materials typically exhibit Ni-based redox activity within the 3.7–4.3 V *vs.* Li/Li^+^ range, with redox peaks progressively shifting toward lower potentials during initial cycles due to electrode activation processes. Ren *et al.*^64^ reported anodic–cathodic peak separations on the order of 0.7–0.8 V for LiNiMnCoO_2_ cathodes during cyclic voltammetry between 2.8 and 4.45 V *vs.* Li/Li^+^, with the voltammograms evolving toward broader redox features centered around ∼3.7 V after repeated cycling.

The moderate electrochemical degradation observed in the present study is consistent with the stabilizing effect of gradient Ni incorporation, which has been reported to mitigate high-voltage structural degradation compared to uniformly modified systems.^65^ Despite the gradual increase in polarization, the persistence of well-defined redox features over 10 cycles indicates that the electroactive structure remains relatively functional under the aggressive operating conditions employed.^21^

Galvanostatic charge–discharge (GCD) measurements were performed at a current rate of 0.5C within a voltage window of 1.0–4.9 V *vs.* Li/Li^+^ to quantify the influence of Ni incorporation on capacity and cycling behavior. Fig. 7c compares the first-cycle GCD profiles of the investigated samples.

The LCO film exhibits strongly sloped charge–discharge curves with no distinct intercalation plateau and a relatively low discharge capacity. This behavior indicates sluggish Li^+^ transport, pronounced polarization, and limited reversible charge storage, which is consistent with the low crystallinity and interfacial limitations typically observed in as-deposited sputtered LCO thin films.^66^ In addition, LCO shows a lower discharge potential (approximately 1 V) compared to the Ni-incorporated samples.

The uniformly Ni-incorporated sample (LNCO-0.25A) exhibits an increased average discharge voltage and higher initial capacity relative to LCO. This improvement can be attributed to the activation of Ni-related redox processes and enhanced electronic conductivity. However, the charge–discharge curves still display broad plateaus and significant voltage hysteresis, indicating persistent polarization and incomplete electrochemical reversibility. These features are consistent with the chemically heterogeneous and hydroxide-rich surface layer identified by XPS and Raman analyses.

In contrast, the gradient Ni-incorporated sample (LNCO-G) delivers the highest initial capacity (∼100–120 mAh g^−1^ at 0.5C) and exhibits a more extended quasi-plateau above ∼3 V, along with a reduced charge–discharge gap. These characteristics indicate lower polarization, more homogeneous Li^+^ transport, and more efficient utilization of the active material. This behavior is consistent with the improved peak definition and reduced peak separation observed in cyclic voltammetry, supporting the interpretation of enhanced electrochemical kinetics in the gradient-structured film.^67^

This improved electrochemical behavior is consistent with the structural and surface analyses, which indicate enhanced local structural ordering in the LNCO-G sample (Fig. 1) and a less hydrated near-surface chemistry, as inferred from XPS results (Fig. 6). Such features are generally beneficial for thin-film cathodes, as a well-ordered layered structure is known to facilitate Li^+^ diffusion and reduce polarization losses, while a more stable interfacial chemistry can mitigate side reactions and structural degradation at high voltage.^66,68^

The NiO- and CoO-related secondary phases are not considered electrochemically inert. NiO can participate in interfacial charge-transfer processes, and CoO may also contribute to the redox response. Therefore, the improved electrochemical response of LNCO-G reflects the combined contributions of the layered phase, the NiO-rich near-surface region, the compositional gradient, and the denser columnar microstructure, rather than the layered phase alone.

It is important to clarify the origin of the apparent excess first-discharge capacity observed in the GCD profiles of LNCO-G within the extended 1.0–4.9 V window. The first-cycle discharge capacity exceeds the corresponding charge capacity, which appears anomalous at first glance. However, this behavior is well-documented in layered oxide cathodes cycled to low cutoff voltages and is attributed to additional Li^+^ insertion and interfacial/electrolyte-related contributions occurring below approximately 2 V.^69^ The extended low-voltage window promotes additional Li^+^ insertion into the layered structure and at interfacial regions, as well as electrolyte decomposition and surface film formation, all of which contribute to the apparent excess capacity. The subsequent sloping profile observed during cycling is consistent with progressive polarization and possible amorphization of the active material, as reported for related systems.^70^ Importantly, the first-cycle capacity is not interpreted as intrinsic reversible capacity, and the low-voltage region is not representative of practical cathode operation.

Fig. 7d presents the charge–discharge curves of the LNCO-G sample recorded during the first and tenth cycles. A moderate increase in voltage hysteresis is observed over cycling, with the electrode retaining approximately 55% of its initial discharge capacity after 10 cycles. This behavior indicates that capacity fading under repeated high-voltage operation is primarily governed by progressive polarization rather than abrupt structural failure.

These observations are consistent with the structurally preserved layered framework and the reduced contribution of reactive surface species suggested by XPS depth profiling. The gradient Ni incorporation therefore appears to contribute to more stable electrochemical behavior by improving both bulk structural ordering and interfacial properties. Such combined effects facilitate Li^+^ transport and contribute to the relatively stable discharge profile observed in the GCD curves.^71^

The specific capacity of ∼100 mAh g^−1^ at 0.5C and its corresponding retention are consistent with performance trends reported for Ni-rich layered cathodes, where capacities on the order of ∼100 mAh g^−1^ are typically achieved at comparable rates, with higher values reached at lower current densities.^72^ These results suggest a relatively reduced degree of polarization and a more reversible electrochemical response compared to the uniformly Ni-incorporated counterpart, in agreement with the observed improvements in charge–discharge profiles and cyclic voltammetry behavior.^73^

Fig. 7e presents the post-cycling Nyquist plots of LCO, LNCO-0.25A, and LNCO-G. Each spectrum contains a depressed semicircle in the high-to-medium-frequency region, assigned to the distributed interfacial charge-transfer response, followed by an inclined low-frequency tail associated with solid-state Li^+^ diffusion.^74^ The high-frequency intercept on the real axis corresponds to the series resistance (*R*_s_), which includes contributions from the electrolyte, electrical contacts, and substrate.

The spectra were fitted using the Randles-type circuit described above. The fitted *R*_s_, constant phase element (CPE) magnitude (*Q*), CPE exponent (*n*), charge-transfer resistance (*R*_ct_), and Warburg coefficient (*σ*) are summarized in Table 3. The obtained values are comparable to those reported in the literature.^75^ Because *R*_s_ combines several cell contributions, differences in *R*_s_ should not be assigned exclusively to film adhesion or contact resistance. Similarly, *σ* is interpreted here as a comparative metric diffusion-related impedance rather than a direct quantitative measurement of the absolute lithium-ion diffusion coefficient (*D*_Li^+^_). Although *D*_Li^+^_ is inversely proportional to *σ*^2^, precise quantitative determination of diffusion coefficients as a function of state of charge requires techniques such as galvanostatic intermittent titration technique (GITT) or scan-rate-dependent CV, which remain subjects for future investigation. Nevertheless, the fitted parameters provide direct kinetic support for the observed electrochemical trends.^76,77^

**Table 3:** Fitted impedance parameters of LCO, LNCO-0.25A, and LNCO-G after electrochemical cycling

Sample	*R*_s_ (Ω)	*Q* (10^−5^ S s^*n*^)	*n*	*R*_ct_ (Ω)	*σ* (Ω s^−1/2^)
LCO	22.4	1.24	0.81	323.5	85.4
LNCO-0.25A	13.8	2.85	0.84	164.3	44.1
LNCO-G	8.5	5.12	0.88	67.7	21.3

LNCO-G exhibits the lowest *R*_s_ (8.5 Ω) and *R*_ct_ (67.7 Ω), corresponding to reductions in *R*_ct_ of approximately 59% relative to LNCO-0.25A and 79% relative to LCO. Its Warburg coefficient is likewise lower (21.3 Ω s^−1/2^) than those of LNCO-0.25A (44.1 Ω s^−1/2^) and LCO (85.4 Ω s^−1/2^), indicating a smaller diffusion-related impedance under the tested conditions. Furthermore, the CPE exponent increases from 0.81 for LCO and 0.84 for LNCO-0.25A to 0.88 for LNCO-G, suggesting a less dispersive and more homogeneous interfacial response. Together, these results show that the gradient film retains a less resistive electrode/electrolyte interface after cycling and support the interpretation that higher structural ordering and reduced surface hydroxyl species facilitate interfacial charge transfer and Li^+^ transport. However, the impedance data alone do not independently establish strain relaxation, crack suppression, or cathode electrolyte interphase thickness. These mechanisms would require post-cycling structural imaging and surface-sensitive interphase characterization.

These findings support a structure–chemistry–kinetics relationship in which gradient Ni incorporation is associated with higher structural order, modified surface reaction products, and reduced post-cycling impedance. A mechanism consistent with the available data involves three coupled effects. First, the higher crystallinity and well-resolved Raman modes of LNCO-G indicate a more ordered transport framework. Second, XPS, Raman, and EDS analyses show a lower concentration of hydroxyl-rich surface species than in LNCO-0.25A, which is consistent with a less reactive interphase. Third, the depth-dependent Ni distribution creates chemically non-equivalent redox environments, as reflected by the multiple partially overlapping CV features. These effects can collectively distribute the electrochemical response over a broader potential range and reduce local polarization.

Taken together, the CV, GCD, and EIS results indicate that the superior response of LNCO-G is likely associated with its spatial Ni distribution rather than Ni incorporation alone. Relative to LNCO-0.25A, LNCO-G combines higher voltammetric current density with a 59% reduction in post-cycling *R*_ct_ and a 52% decrease in *σ*. These quantitative differences provide direct evidence of reduced interfacial and diffusion-related impedance in the gradient coating.

Finally, it is important to clarify the scope of the electrochemical protocol. The present work is a materials-level comparison of as-deposited thin films, not a cell-engineering or lifetime study. The 10-cycle test at 0.5C within 1.0–4.9 V was intentionally chosen as a short, aggressive screening protocol to amplify differences in polarization, interfacial stability, and early capacity retention among the three architectures. Under this extended window, prolonged cycling would be dominated by electrolyte decomposition, surface-film growth, and substrate/current-collector effects rather than by the intrinsic structural response of the Ni-gradient film. Rate-capability measurements at multiple current densities and extended cycling over 50–100 cycles would require a dedicated cell-optimization campaign, including stable reference/counter electrodes, electrolyte formulation, and reproducible assembly, which lies beyond the comparative scope of this study. Moreover, because LNCO-0.25A and LNCO-G already differ in total Ni content and secondary-phase fraction, additional cycling data alone would not isolate the gradient effect without a composition-matched control. The 10-cycle comparison is therefore reported as a preliminary but internally consistent indicator of the relative electrochemical response. Extended cycling, rate-capability tests, and composition-matched controls are identified as priority future work to establish practical performance limits.

## 4. Conclusions

The present work demonstrates that tailoring the Ni incorporation strategy in RF-sputtered LiCoO_2_-based thin films provides a route to tune structural properties, surface chemistry, and electrochemical performance. Three architectures were investigated: LCO, LNCO-0.25A, and LNCO-G, the latter achieved by progressively increasing the Ni target current during co-sputtering.

Structural characterization by XRD and Raman spectroscopy indicates that Ni incorporation is accompanied by improved local structural ordering, with the most pronounced effect observed for the gradient architecture. Cross-sectional HAADF-STEM and STEM–EDS mapping directly confirm a dense microstructure and a continuous Ni gradient in LNCO-G, with Ni increasing progressively from the substrate interface toward the outer surface. XPS depth profiling reveals that, although all samples develop carbonate-rich surface layers upon exposure to air, gradient Ni incorporation leads to a reduced contribution of hydroxylated species and a less hydrated near-surface chemistry compared with the uniformly incorporated counterpart.

Electrochemical measurements highlight the influence of Ni incorporation strategy on performance under high-voltage conditions. While LCO exhibits low current density, pronounced polarization, and limited capacity, and uniform Ni incorporation improves initial activity but remains hindered by surface reactivity, the gradient Ni-incorporated LNCO-G sample delivers the most favorable response. In particular, LNCO-G exhibits higher current densities (≈3–5× increase relative to LNCO-0.25A), improved peak definition, and a first-cycle discharge capacity of ∼100–120 mAh g^−1^ at 0.5C. Post-cycling EIS corroborates these trends: LNCO-G exhibits *R*_ct_ = 67.7 Ω and *σ* = 21.3 Ω s^−1/2^, corresponding to reductions of approximately 59% and 52%, respectively, relative to LNCO-0.25A.

The persistence of well-defined redox features and retention of ∼55% capacity after 10 cycles under these aggressive conditions; a protocol purposely chosen as a materials-level screening test; indicate moderate degradation under severe conditions rather than practical long-term stability.

Taken together, the superior response of LNCO-G is associated with its spatial distribution, which is accompanied by improved structural order, reduced hydroxyl-rich surface species, and lower post-cycling interfacial and diffusion-related impedance. These observations support gradient Ni incorporation as a promising route for tuning coupled structure–surface–kinetics relationships in sputtered LiCoO_2_ thin-film cathodes.

However, because LNCO-0.25A and LNCO-G differ in total Ni content and secondary-phase fraction, the present comparison cannot isolate the effect of the Ni gradient alone; a composition-matched uniform control is required to decouple these contributions and is identified as future work.

Future work will also focus on quantifying absolute Li^+^ diffusion coefficients using GITT and scan rate-dependent CV, extending cycling to 100 cycles and performing rate-capacity tests, alongside higher-resolution depth profiling and post-cycling interphase characterization.

## Author contributions

Conceptualization, Z. B., J. D. C., and S. M. A. C.; methodology, Z. B., J. D. C., S. M. A. C., and S. C.; validation, Z. B., J. D. C., S. M. A. C. and S. C.; formal analysis, Z. B., C. V., J. D. C., and S. M. A. C.; investigation, Z. B., C. V., J. D. C., and S. M. A. C.; resources, S. C., and S. M. A. C.; data curation, Z. B., C. V., and J. D. C.; writing – original draft preparation, Z. B. and C. V.; writing – review and editing, Z. B., J. D. C., S. M. A. C., and S. C.; visualization, Z. B., J. D. C., S. M. A. C., and S. C.; supervision, Z. B. and S. C.; funding acquisition, S. M. A. C. All authors have read and agreed to the published version of the manuscript.

## Conflicts of interest

There are no conflicts to declare.

## Supplementary Material

RA-OLF-D6RA06673A-s001

## Data Availability

Data for this study, including figures and tables are available at article. The data supporting this article have been included as part of the supplementary information (SI). Supplementary information: detailed deconvoluted Raman spectra and peak fitting parameters (positions, FWHM, and relative intensity ratios for LCO, LNCO-0.25A, and LNCO-G thin films), as well as core-level XPS binding energy and FWHM fitting parameters (C 1s, Li 1s, O 1s, Co 2p, and Ni 2p). See DOI: https://doi.org/10.1039/d6ra06673a.
