# Supplementary material for: Influence of gradient Ni incorporation on the structural, surface, and electrochemical properties of LiCoO2 thin-film cathodes
Source: RSC Adv. 2026 Oct 22. Online ahead of print. doi: 10.1039/d6ra06673a (PMC13690019; doi:10.1039/d6ra06673a)
Supplement: Supplementary file 1 [file RA-OLF-D6RA06673A-s001.pdf]

Supplementary Information (SI) for RSC Advances.
This journal is © The Royal Society of Chemistry

Supplementary information

Supplementary Figure:

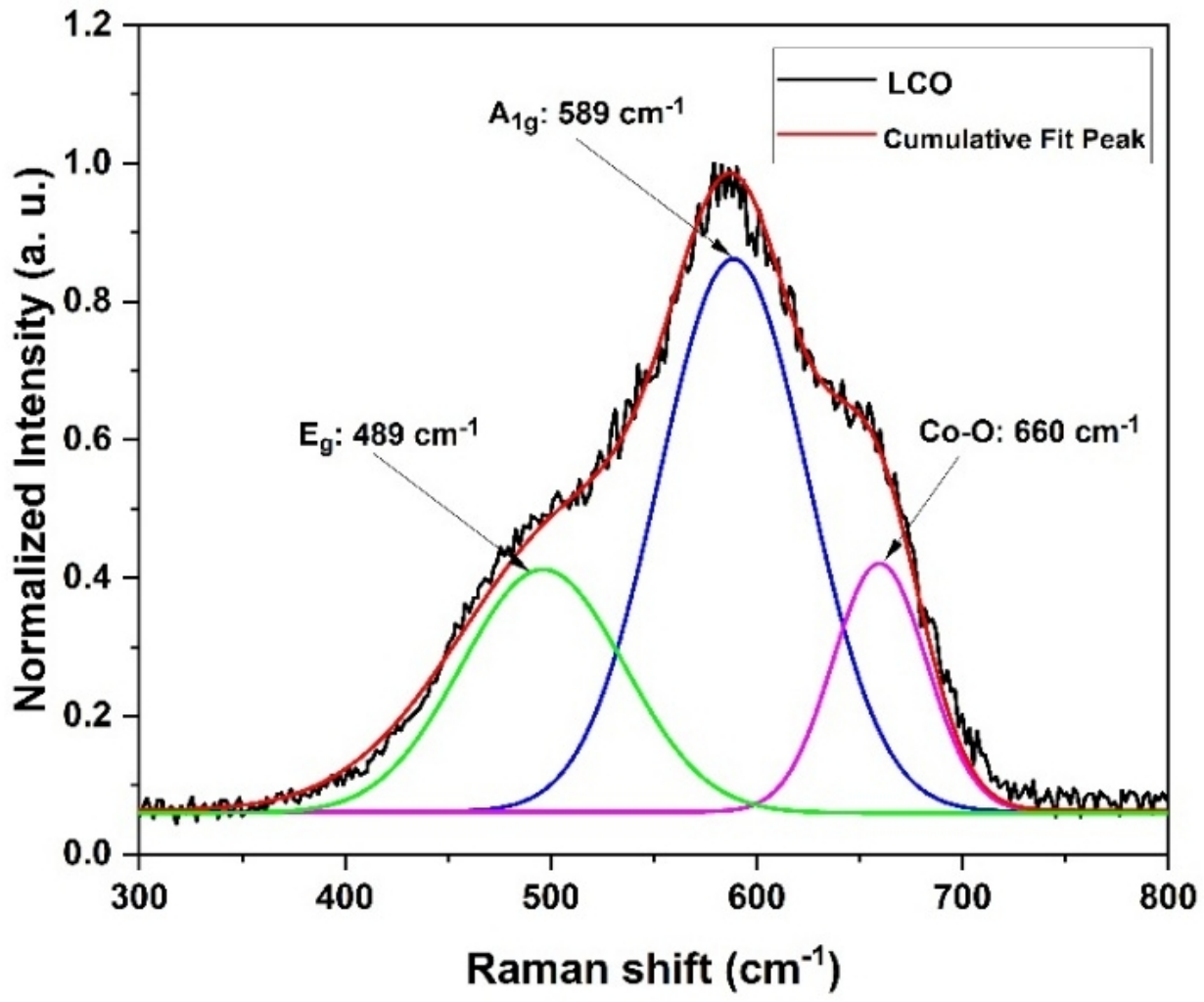

(a)

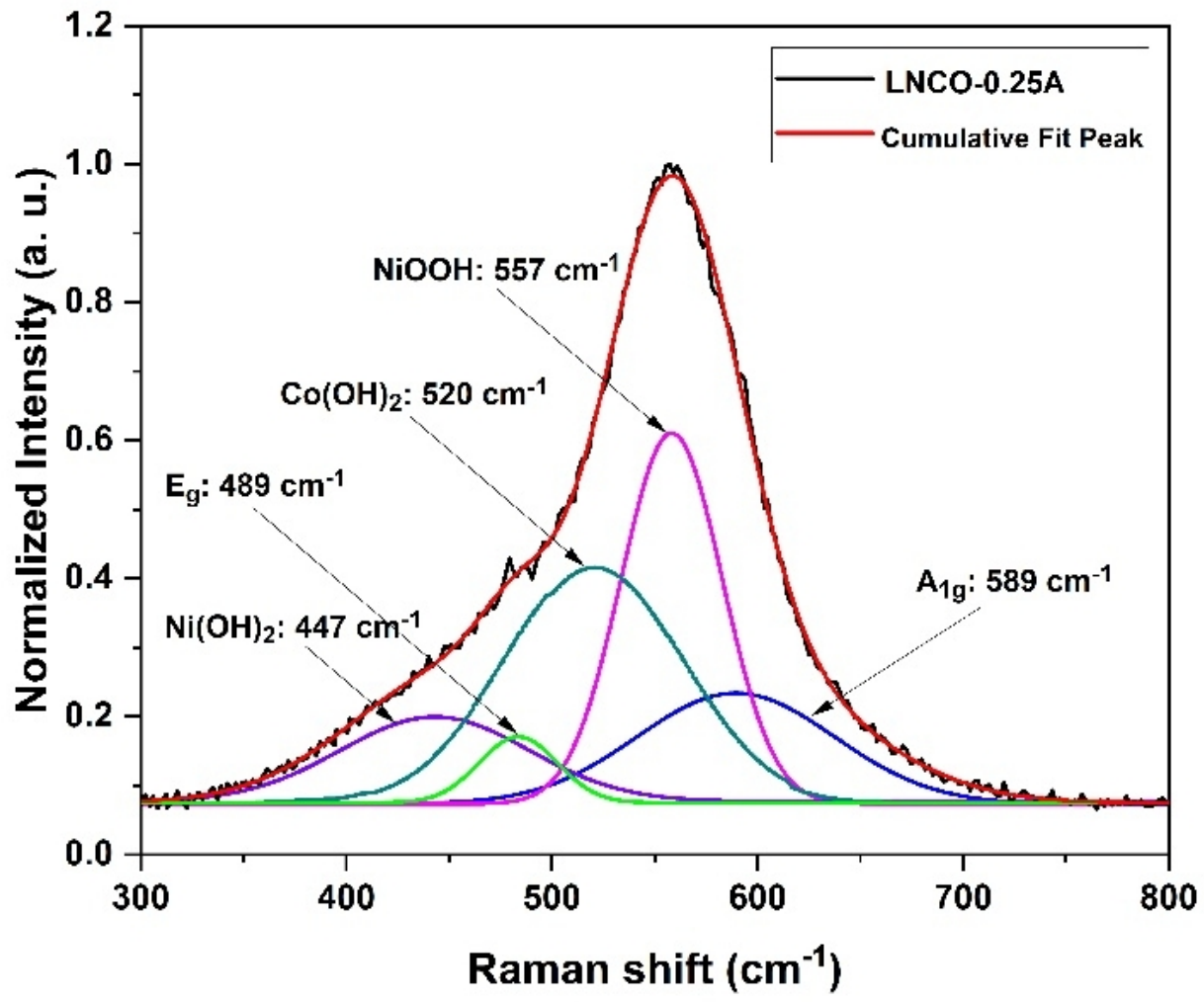

(b)

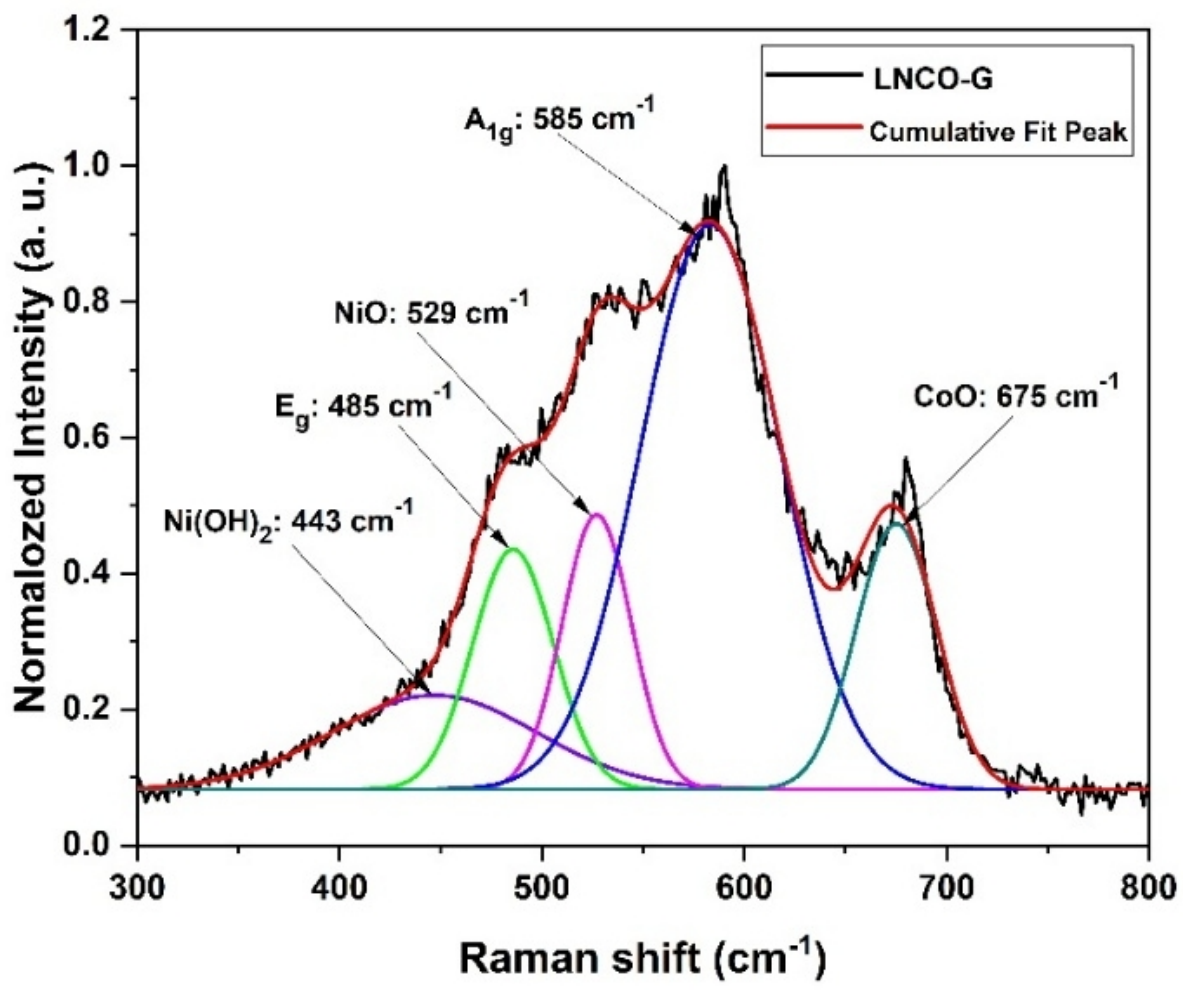

(d)

Figure S1. Curve-fitting of Raman spectrum for the LCO (a), LNCO-0.25A (b), and LNCO-G (c) coatings.

Supplementary Tables

Table S1: Raman spectral parameters, including peak positions, full width at half maximum (FWHM) values, and relative intensity ratios, for LCO, LNCO-0.25A, and LNCO-G thin films.

Sample	Peak	Peak Positions (cm^{-1})	FWHM (cm^{-1})	$I(A_{1g})/I(E_g)$	$I_{phase}/I(A_{1g})$
LCO	E_g	489	104.9	1.9	-
	A_{1g}	589	89.3		-
	CoO	660	59.6		0.7
LNCO-0.25A	E_g	489	79.6	1.4	-
	A_{1g}	589	154.55		-
	$Ni(OH)_2$	447	167.1		0.8
	$Co(OH)_2$	520	118.9		1.8
	NiOOH	557	63.1		2.6
LNCO-G	E_g	485	54.9	2.1	-
	A_{1g}	585	86.9		-
	$Ni(OH)_2$	443	180.2		0.2
	NiO	529	46.4		0.5
	CoO	675	53.9		0.5

Table S2: XPS fitting parameters of core levels.

Label	Core level	BE (eV)	FWHM (eV)

C-O_3	C 1s	290.1 ± 0.2	1.5 ± 0.2
C-O-H	C 1s	286.5 ± 0.1	2.7 ± 0.6
(Li/)LiOH/Li_2CO_3	Li 1s	55.0 ± 0.4	1.9 ± 0.3
Li_2O	Li 1s	56.4 ± 0.3	1.4 ± 0.7
OH/CO_3	O 1s	531.8 ± 0.3	1.9 ± 0.1
O	O 1s	528.9 ± 0.7	1.8 ± 0.1
CoO	Co 2p	779.7 ± 0.3	2.2 ± 0.1
CoOOH	Co 2p	780.3 ± 0.5	1.4 ± 0.2
$Co(OH)_2$	Co 2p	781.3 ±0.4	1.6 ±0.3
Ni	Ni 2p	851.6 ± 0.3	1.3 ± 0.1
NiO	Ni 2p	853.8 ± 0.4	2.9 ± 0.2
$Ni(OH)_2$	Ni 2p	855.8 ± 0.3	3.7 ± 0.6
